# *Toxoplasma* Modulates Signature Pathways of Human Epilepsy, Neurodegeneration & Cancer

**DOI:** 10.1038/s41598-017-10675-6

**Published:** 2017-09-13

**Authors:** Huân M. Ngô, Ying Zhou, Hernan Lorenzi, Kai Wang, Taek-Kyun Kim, Yong Zhou, Kamal El Bissati, Ernest Mui, Laura Fraczek, Seesandra V. Rajagopala, Craig W. Roberts, Fiona L. Henriquez, Alexandre Montpetit, Jenefer M. Blackwell, Sarra E. Jamieson, Kelsey Wheeler, Ian J. Begeman, Carlos Naranjo-Galvis, Ney Alliey-Rodriguez, Roderick G. Davis, Liliana Soroceanu, Charles Cobbs, Dennis A. Steindler, Kenneth Boyer, A. Gwendolyn Noble, Charles N. Swisher, Peter T. Heydemann, Peter Rabiah, Shawn Withers, Patricia Soteropoulos, Leroy Hood, Rima McLeod

**Affiliations:** 1 The University of Chicago, Chicago, IL 60637 USA; 2 Northwestern University, Feinberg School of Medicine, Chicago, IL 60611 USA; 3 BrainMicro LLC, New Haven, CT 06511 USA; 4 J Craig Venter Institute, Rockville, MD 20850 USA; 5 Institute for Systems Biology, Seattle, WA 98109 USA; 6 University of Strathclyde, Glasgow, G1 1XQ United Kingdom; 7 Genome Quebec, Montréal, QC H3B 1S6, Canada; McGill University, Montréal, QC H3A 0G4 Canada; 8 Department of Pathology, University of Cambridge, Cambridge, CB2 1QP United Kingdom; 9 Telethon Kids Institute, The University of Western Australia, Perth, Australia; 10 University of Illinois-Chicago, Chicago, IL 60607 USA; 11 California Pacific Medical Center, San Francisco, CA 94114 USA; 12 JM USDA Human Nutrition Research Center on Aging, Tufts University, Boston, MA 02111 USA; 13 Rush University Medical Center, Chicago, IL 60612 USA; 14 Northshore University Health System, Evanston, IL 60201 USA; 15 Rutgers University, Newark, New Jersey 07101 USA; 16 FLH, IBEHR School of Science and Sport, University of the West of Scotland, Paisley, PA1 2BE UK

**Keywords:** Disease genetics, RNA sequencing, Predictive markers, Microbiology

## Abstract

One third of humans are infected lifelong with the brain-dwelling, protozoan parasite, *Toxoplasma gondii*. Approximately fifteen million of these have congenital toxoplasmosis. Although neurobehavioral disease is associated with seropositivity, causality is unproven. To better understand what this parasite does to human brains, we performed a comprehensive systems analysis of the infected brain: We identified susceptibility genes for congenital toxoplasmosis in our cohort of infected humans and found these genes are expressed in human brain. Transcriptomic and quantitative proteomic analyses of infected human, primary, neuronal stem and monocytic cells revealed effects on neurodevelopment and plasticity in neural, immune, and endocrine networks. These findings were supported by identification of protein and miRNA biomarkers in sera of ill children reflecting brain damage and *T. gondii* infection. These data were deconvoluted using three systems biology approaches: “Orbital-deconvolution” elucidated upstream, regulatory pathways interconnecting human susceptibility genes, biomarkers, proteomes, and transcriptomes. “Cluster-deconvolution” revealed visual protein-protein interaction clusters involved in processes affecting brain functions and circuitry, including lipid metabolism, leukocyte migration and olfaction. Finally, “disease-deconvolution” identified associations between the parasite-brain interactions and epilepsy, movement disorders, Alzheimer’s disease, and cancer. This “reconstruction-deconvolution” logic provides templates of progenitor cells’ potentiating effects, and components affecting human brain parasitism and diseases.

## Introduction

The first half of the 20^th^ century achieved remarkable advances in control of some communicable diseases, with development of immunizations, antimicrobial therapies, and increasing ability to identify new pathogenic organisms^1^. The second half shifted to understanding chronic degenerative diseases as prevailing causes of death in older populations. One primary challenge for contemporary medicine is to control non-communicable diseases with complex gene-environment etiologies and progression^2, 3^, postulated to arise from complex interactive cascades of genetic and environmental factors. Historical efforts to find causes and cures for such illnesses, including brain diseases, have had a glaring omission of a significant environmental factor: Over 2 billion humans are infected with the neurotrophic parasite *Toxoplasma gondii*. Congenital and postnatal infections with *T. gondii* persist in all infected persons. The parasite interconverts between slow-growing, encysted bradyzoites and rapid-growing tachyzoites^4^. In mice, *T. gondii* creates a chronic intra-neuronal infection and an inflammatory process^4^. Mice with acute and chronic infection have alterations in neurotransmitters, memory, seizures, and neurobehaviors^5, 6^. Some epidemiologic-serologic studies show associations between seropositivity for *T. gondii* and human neurologic diseases, for example, Parkinson’s and Alzheimer’s diseases^7, 8^. Serologic studies of humans with diverse genetics are not optimal to detect strong associations or directionality. Epidemiologic associations also do not reveal parasite-modulated gene networks in human brain that could provide insights into how to cure and prevent resultant diseases. We need integrative approaches to examine relationships between brain parasitism and other brain diseases^9, 10^, to provide a foundation to identify key pathways and molecules for drug and vaccine design.

To address these problems, we considered two central questions: (i) If chronic brain parasitism associates with other neurologic diseases, what are they? and (ii) Which macromolecular networks are modulated by the parasite in human brain that lead to neuropathology which could underpin and facilitate design of treatments? We hypothesized that a systems approach integrating multiple levels of host parasite interactions might resolve these questions. To gain insights into relationships between human *T. gondii* infections and brain disorders, we studied a unique cohort, the National Collaborative Chicago Based Congenital Toxoplasmosis Study (NCCCTS), and identified human susceptibility genes, as well as serologic biomarkers of active brain disease. The NCCCTS has diagnosed, treated and followed 246 congenitally infected persons and their families continuously beginning in 1981^11–39^. Next in our study, we obtained new transcriptomic and proteomic data from infections of primary, human, neuronal stem cells, and monocytic cells that infiltrate brain, to determine whether there are different phenotypic effects of Type I, II, or III *T. gondii* tachyzoites. We used these four data sets to construct an integrated molecular model of the infected human brain. In a second phase, the model was further analyzed using systems biology approaches to provide insights into the molecular mechanisms by which *T. gondii* infection may cause disease. The broader goal was to provide a robust database and informatic analysis for the scientific community to use for their research. This report described only a limited number of important observations. This effort to integrate multiple levels of intrinsic and extrinsic factors offers an original template to unravel pathogenesis of complex diseases in humans.

**Figure 1:**
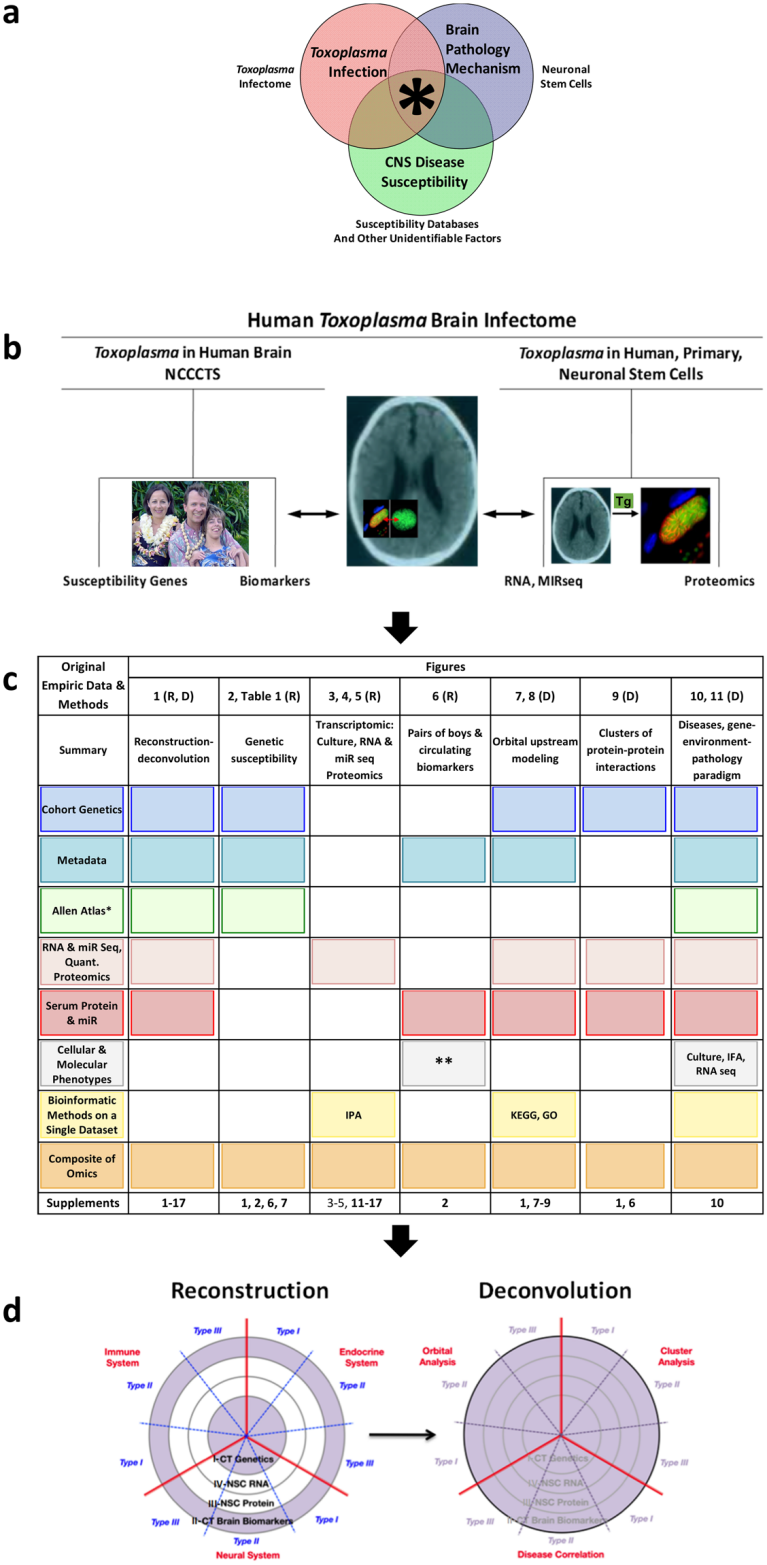
Methodology and analyses for understanding interaction of *Toxoplasma gondii* with human brain. (**a**) Gene-environment-pathology paradigm. The Venn diagram shows model of pathogenesis with confluence of permissive host and parasite genetics, and exposure. (**b**) Flow diagram of empirical genetic and biomarker data from NCCCTS, transcriptomics and proteomics. (**c**) Structure of the manuscript. This includes original empiric data, methods for analyses, and contributions of components to analyses in each figure. *Empiric but not from NCCCTS cohort; **Cell culture, IFA, microarray gene expression, mRNAseq, miRseq, quantitative proteomics, miR qPCR. d. Reconstruction and deconvolution analyses. Reconstruction is the discovery, integration and systems analysis of interrelatedness of four areas of primary, original data: genetics, transcriptomics and proteomics of infected cells and circulating serum biomarkers in ill persons. Deconvolution refers to the systems analysis that examines upstream regulatory genes, protein-protein cluster interactions and diseases with which biosignature pathways associate. These are the topics of the current work and are elaborated on throughout this manuscript. Image of family reproduced with their permission and also from “The Billion Brain Parasite”, *Science Life* (Easton, 2014).

Our studies presented here were performed to examine genetic and parasite effects we hypothesized would influence outcomes (Fig. 1a). We utilized a novel systems approach expanding from gene-environment paradigms to include a third component of development (Fig. 1a). In Fig. 1a, there is intersection/overlap between the circles representing components of the host-parasite interactions. These circles include host-parasite genetics, toxoplasmosis susceptibility genes, and serum biomarkers in children in the NCCCTS [*T. gondii* infection, red circle], human neuronal stem cell functional assays including transcriptomics and proteomics [Brain pathology mechanisms, blue circle], and disease pathogenesis/pathology susceptibility genes for other diseases [others, green circle]). This overlap/intersection in the Venn diagram indicates the circumstances in which we hypothesize that manifestations of other diseases will occur. To test this hypothesis, we isolated the infected brain as a system. This sequence of our work presented herein, and structure of our studies, and these results are shown in a flow chart (Fig. 1b), detailed outline (Fig. 1c), and schematic diagram of our model created based on this work (Fig. 1a and d). The overall plan was to gain access to the network interactome and biosignatures of *T. gondii* and the human brain, which first reconstructed the *T. gondii* brain infection. Our first steps of reconstructing *T. gondii* brain infection included discovery, integration and systems analysis of our original data. These systematic analyses of our novel human data sets used empiric studies of human *T. gondii* infections of persons with toxoplasmosis and their families, and infectomes of primary, human brain stem and monocytic cells (Fig. 1b,c). In our work, the human infectome is defined as the human host and parasite molecules, and pathways that are perturbed by the interaction of the human host and parasite *T. gondii*, as has been characterized by others for studies of other pathogens in earlier literature. An interactome is the whole set of molecular interactions such as protein-protein and genetic interactions. This can provide a global “Omic” view of molecular effects as in the resource APID interactome (http://cicblade.dep.usal.es:8080/APID/init.action). To our knowledge, this study herein is the only single human cohort that is directly observed by a uniform group of examiners, longitudinally in a variety of ways, combined with human, primary, neuronal stem cell Omics Systems analysis for the *T. gondii* infectome, then interrogated for disease susceptibility genes/proteins. We obtained cellular data to test a few pathways relevant to pathogenesis. These first were considered individually and then together to determine whether we identified key biologic processes and biosignatures affected by *T. gondii*. Second, the integrated brain infectome became a global map that was deconvoluted to determine functional clusters and disease correlates (Fig. 1d). We termed this approach “Reconstruction and Deconvolution” (Fig. 1d). Specifically, Reconstruction was based on our unique cohort of infected persons, most of whom showed neuropathologic symptoms^13^, to identify novel human genetics of susceptibility to toxoplasmosis. Then, serologic biomarkers were studied for a limited number of infected humans to assay readout of an infected brain. We selected neural stem cells to uncover potential developmental mechanisms because of their multipotency central to neurodevelopment and neuroplasticity. Most brain diseases result from abnormal neurodevelopment (e.g., epilepsy) and neuroplasticity (e.g., neurodegeneration). Hence, transcriptomic and proteomic infectomes of human neural stem cells were studied for parasite effects using primary cultures of cells from the hippocampus-temporal lobe. These datasets were integrated into a total brain infectome. They were unraveled, or “deconvoluted”, to identify functional and disease correlates. This was accomplished by analyzing upstream regulators in all our datasets. Thereby, we determined how different brain infectome components were interrelated. Cluster protein-protein interaction analysis revealed additional, functional correlates. Associations between the *T. gondii* brain infectome and other diseases that share these signature interactions were determined using our empirical, primary data.

## Results

### Reconstruction and deconvolution of *Toxoplasma gondii* brain infection

#### Reconstruction 1. Susceptibility genes expressed in human brain provide insight into signature pathways of *T. gondii* in human brain

**Figure 2:**
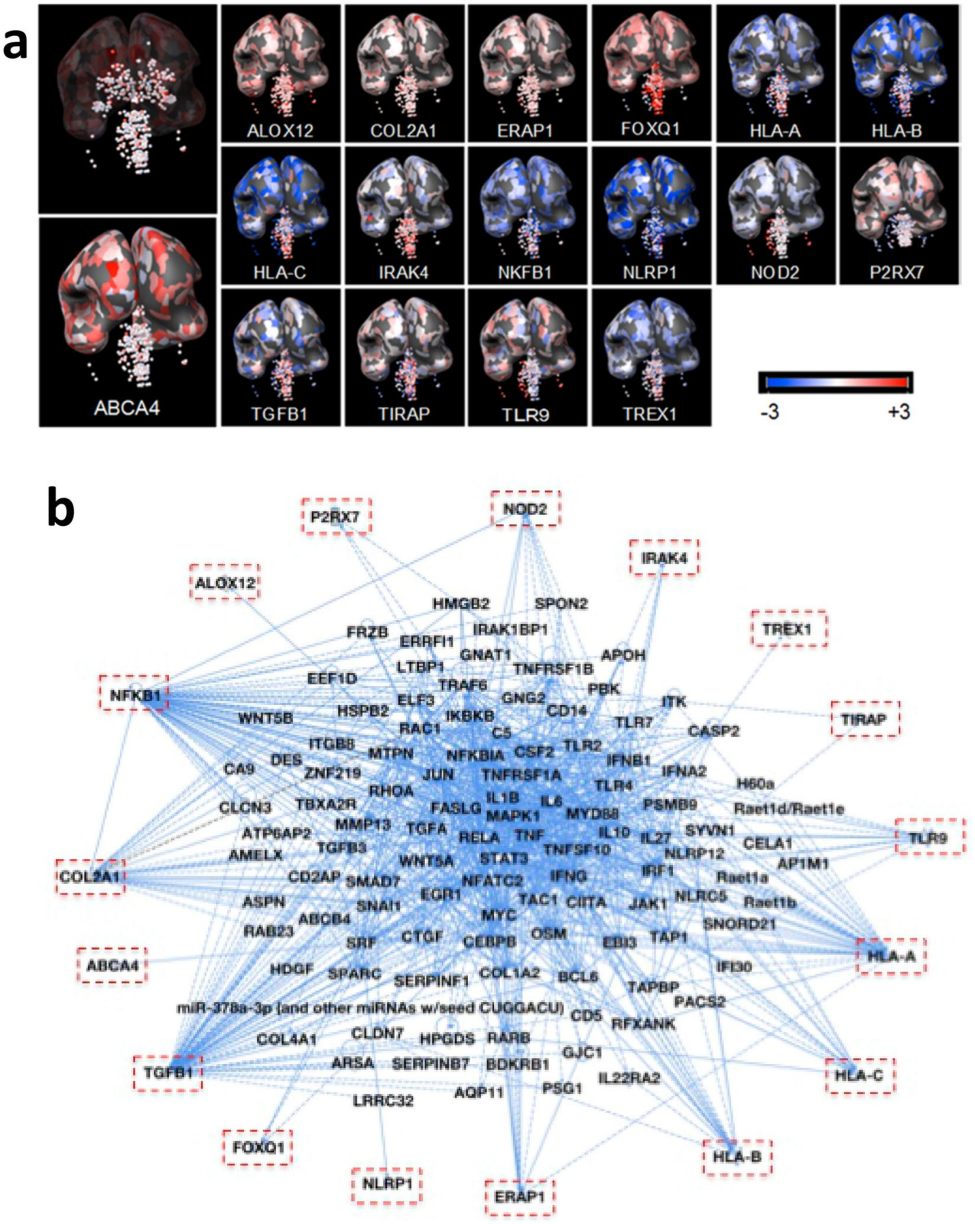
Susceptibility genetics. (**a**) Expression and localization in human brain utilizing Allen Brain Atlas of genes with alleles conferring susceptibility to congenital toxoplasmosis in National Collaborative Chicago Based Congenital Toxoplasmosis Study (NCCCTS). Transcript expression is visualized for brain of the youngest donor (H0351.2001, 24 years, male, African-American). Z-score of microarray data ranges between −3 and +3 to quantify the lowest to highest expression (see Supplement B: Table S2). (**b**) Ingenuity Pathway Analysis (IPA) of NCCCTS susceptibility genes and upstream regulators. Cut off of p value at 5 × 10^−3^ generated a network of 117 predicted upstream regulators (see Supplement B: Table 2). Upstream regulators and susceptibility genes are consolidated/bundled and graphically mapped. Susceptibility genes are manually relocated in IPA graphic to show connectivity to upstream regulators. Note NFκB and TGFβ are central nodes that are visual.

Our unique NCCCTS cohort of persons with congenital toxoplasmosis and their families have been carefully characterized longitudinally^13, 40–44^ (Fig. 1). Ongoing evaluations of this cohort contributes to the first phase of our present analyses as shown in the flow diagram in Fig. 1a. Figure 1b presents an outline of the work in this study including how the genetic and cohort analyses form a basis for the work. Figure 1c shows that these cohort and genetics analyses are integrated with other aspects of the current work in a model. Our NCCCTS cohort is the source of previously published analyses^11–39^ which earlier provided a powerful tool to identify genes and pathways causing susceptibility to toxoplasmosis (Table 1^11–39^, Figs 1 and 2). These susceptibility genes identified earlier are considered along with susceptibility genes newly identified herein. All these genes are part of our further analyses in this present manuscript. In our earlier work, the human susceptibility alleles of candidate genes identified for those in the NCCCTS were *HLA Class I* and *II genes, ERAP 1, COL2A1*, *ABCA4*, *P2RX7, ALOX12, NALP1, IRAK4* (Table 1). Some of these gene/susceptibility associations were further confirmed using samples from the European Cohort study (EMSCOT)^12, 14^. One association, with NOD2^39^, was present in a Brazilian Cohort with eye disease, but not found in the NCCCTS. Characterization of mechanisms associated with *ERAP1* were extended in studies of cross presentation of antigen^45^. Peptides interacting with MHC Class I genes of greater than usual octamer/nonamer lengths^45^ also were identified, suggesting that *T. gondii* subverts its host’s immune defenses with aberrant splicing of polypeptides^46^. These genetic data and their analysis are summarized in Table 1 and Fig. 2a and b. The newly identified genes or phenotypes identified herein are labeled “AM” or “OD” in Table 1

**Table 1:** Genes with Susceptibility/Resistance Alleles Defined with National Collaborative Toxoplasmosis Study and EMSCOT Cohorts.

Gene	SNP (Allele)	P Value	Reason Candidate Gene	Replicate/Proof of Principle/Phenotype	Reference/Supporting Data
*HLA Class II*	DQ3	<0.02	MD	Hydrocephalous in children (DQ3), Fewer cysts in HLA transgenic mice (DQ1)	11
	DQ1	<0.0005			
*COL2A1*	rs6823 (G)	<0.03 (brain)	ED	EMSCOT replicates, imprinted, brain and eye disease	12, 13
	rs2276455 (A)	<0.03			
	rs2276455 (G)*	<0.0005			
	rs1635544 (C)	<0.03			
	rs2070739 (T)	<0.02			
	rs2276454 (A)	<0.007			
	rs3803183 (T)	<0.02			
	rs3803183 (T)*	<0.003			
*ABCA4*	rs952499 (C)	<0.03	HC	EMSCOT replicates, imprinted, localized in human brain	12, 13, HC
	rs952499 (T)*	<0.005			
	rs2297633 (G)*	<0.0003			
	rs1761375 (G)*	<0.0001			
	rs3112831 (C)*	<0.02			
*P2RX7*	rs1621388(C1772T)	<0.021	OI	EMSCOT replicates for differing alleles; ATP mediated cell death, cytokine signaling, pro-inflammation	14, 15, OD
	rs1718119(T1068C)	<0.015			
*HLA Class I*	A	<0.01	MD	Genotype association and phenotypes humans and mice. PBMC from cohort. Peptides for HLA A2, A11, B7 confer protection	16–22, OD
	B				
	C				
*ERAP1*	rs149173(T/C)	<0.0077	LfL	Genotype association and phenotypes humans and mice	16, OD
	rs17481856(C/T)	0.0253			
*IRAK4*	rs1461567	<0.023	IOID	Genotype; phenotype, cell death, inflammation	23
	rs4251513	<0.045			
*NALP1*	rs8081261	<0.002	MD TRNG	Genotype (MD region; human); phenotype, cell death, inflammation; MD	24–30
	rs11652907	<0.02			
	rs9902174	<0.04			
*ALOX12*	rs6502997	<0.0003	MD TRNG	Genotype and phenotype, cell death proinflammation	31
	rs312462 (C)	<0.03			
	rs6502998 (C)	<0.03			
	rs434473	<0.04			
*TLR9*	rs574386 (T1905C)	<0.008	TLRs	Brazil and Poland replicates; phenotype, ligand	32
	rs352140 (C)	<0.0001			AM
*TIRAP*	rs8177374(S180L)	<0.006	IOID	Genotype; Phenotype TLR signaling and cytokines	AM
*FOXQ1*	rs920209	<0.02	HC	Note NK cells mice	33, OD
*TREX1*	rs 2242150(A/T)	0.02	SPD	Related clinical & Type 1 IFN phenotype, LFL	34, 35, OD or AM
*NFκβ1*	rs997476(C/A)	<0.02	CtoPiEA	Phenotype, nuclear localization, signaling pathway	36, OD
*TGFβ1*	rs10417924 (G overtranscribed)	0.016	CtoPiEA, MD	Phenotype transcriptomics, GRA1	37, 38
*NOD2 (Brazil)*	rs3135499 (C/A)†	<0.04	LfL Brazil	Eye Disease, IL17, CD4+	39

Abbreviations: Reason for selection of gene to test sequentially as single candidate gene in NCCCTS (1981–2016): MD, Murine model data; ED, Eye disease in humans caused by mutation of this gene; HC, Hydrocephalous caused in humans by gene mutation and adult macular degeneration associated with allelic variants; OI, Implicated alleles for other infections; LfL, Logical to test from literature and other findings, for example MHC Class I presentation of antigen for ERAP1; IOID, Gene in other diseases in the literature; MD TRNG, Toxo 1 region, not gene initially in humans based on rat Toxo 1 region; TLRs, Testing TLR genes now replicated by other cohorts and proven to be important in mice; SPD, similar pattern of brain disease as AG brain disease due to DNA ligase mutations; CtoPIEA, Central to genetic pathway identified with original analysis led to TDT analysis herein. Note: LfL Brazil, Minas Gerais, did not replicate in US full cohort; no *, significant in NCCCTS not EMSCOT;* significant in EMSCOT cohort not NCCCTS. P values are nominal. Supporting Data [SD]: References (#) with narrative summary of findings and gene function for published work or in preparation (AM) or original data in this manuscript (OD) in Fig. 1 or online supplement.

. These susceptibility alleles indicated that the candidate genes were playing a role in susceptibility to toxoplasmosis and some have been studied for corresponding phenotypes to explain that susceptibility. The previously described genes are combined with newly identified genes for the analyses herein.

In a preliminary, initial network reconstruction analysis performed with Ingenuity Pathway (IPA), we noted that NFκB and TGFβ were central nodes in the network (McLeod, R, Lorenzi H, *et al*., JCVI White Paper; http://gcid.jcvi.org/docs/Toxoplasma_gondii_06152012.pdf). Therefore, they became predicted candidate susceptibility genes. Using Transmission Disequilibrium Testing (TDT) with DNA from the families in the NCCCTS, we further confirmed herein that *NFkB* and *TGFβ* were actually empirically confirmed human toxoplasmosis susceptibility genes (Table 1, Fig. 2b, Supplement B: Table S1). Our sequential analysis of other candidate susceptibility *loci* presented herein, found that *TREX1, TIRAP MAL, FOXQ*, and *TLR9* also had allelic variants significantly associated with susceptibility to congenital toxoplasmosis in the NCCCTS (p < 0.05) (Table 1).

It is noteworthy that all these identified, critical genes are expressed in the human brain, as shown in red coloration in Fig. 2a. Specifically, expression analysis using the Allen Human Brain Atlas (ABA) followed by 3-D Brain Explorer 2 software analysis revealed that all 17 candidate susceptibility genes are expressed in the human brain^47^. Z-scores of RNA expression of 6 human brains in the ABA database demonstrated localization and increased or decreased expression of these genes in these human brains (Fig. 2a) in the hippocampus, choroid plexus, and globus pallidus.

Our genetic analyses provided a foundation for systems analysis of congenital toxoplasmosis as it affects the human brain (Figs 2b, S2). Upstream regulators of all these genes also were identified: a network of our susceptibility genes and upstream regulators was constructed with IPA (p-value of overlap <5 × 10^−3^; Fig. 2b) to identify critical connecting nodes (genes). *IFNϒ, TLR4, TAP 1, JUN, MYC, TNFR* were identified as upstream regulators, among others (Fig. 2b). Among 112 upstream regulators of these genes (Fig. 2b), 96 mediate both cellular growth and proliferation (p = 5.22 × 10^−35^) and cell death and survival (p = 3.26 × 10^−37^; Supplement B: Table S2).

#### Reconstruction 2: *T. gondii* modulates human brain stem cell transcriptomes and proteomes

**Figure 3:**
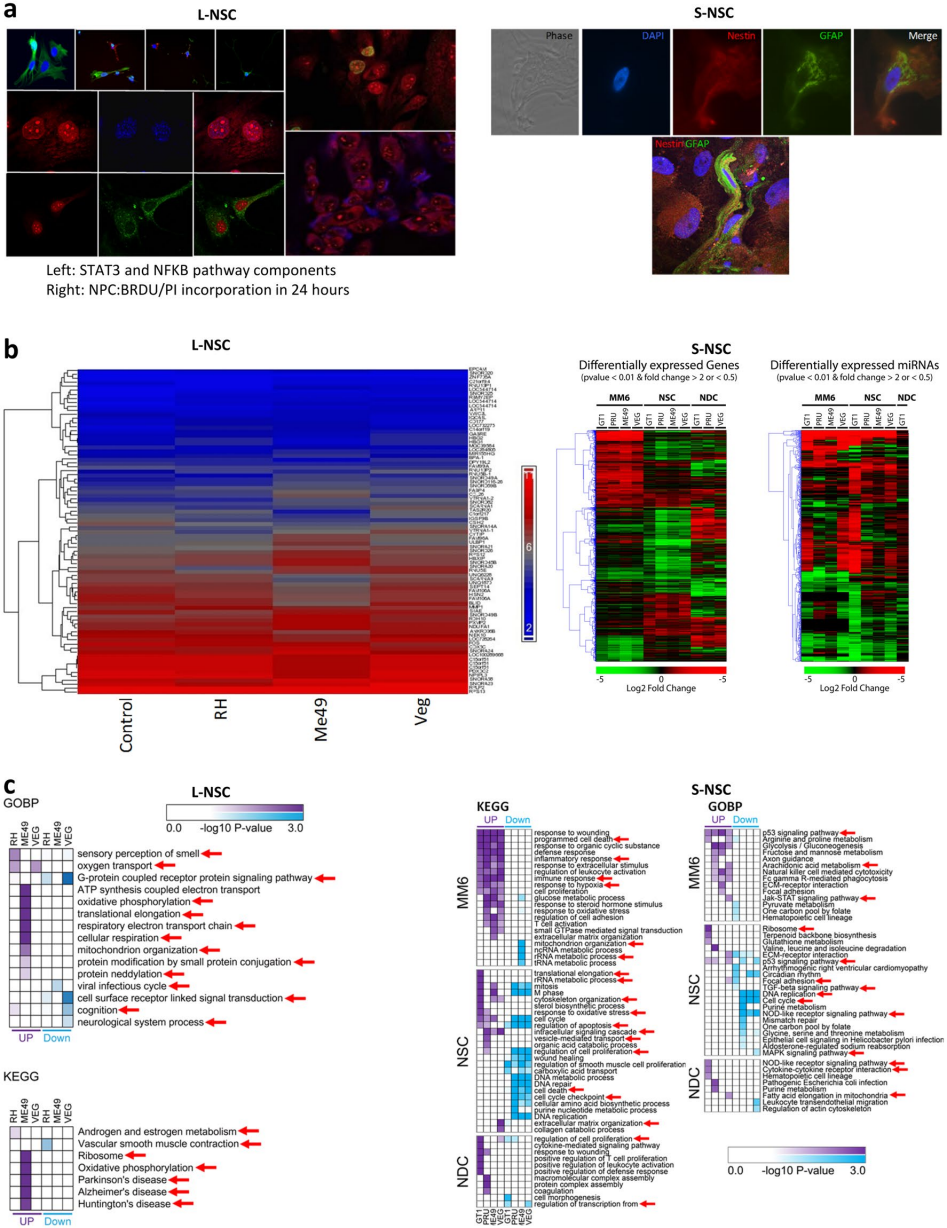
Transcriptomics and their Analyses. (**a**) Immunostaining of L-NSC and S-NSC cell lines. Left panel in a shows L-NSC cells stained for nestin (green) and Tuj1 (red) (upper row, 20X), Stat 3 (middle row, red) and NFκB (lower row, green), nuclear counterstain blue, DAPI (40X). The right side of the left panel in a shows L-NSC cells immunostained for the cell proliferation marker bromodeoxyuridine, BrdU(upper panel), and propidium, PI (lower panel), nuclear counterstained, DAPI blue (40X). A, right panel, phase microscopic image of S-NSC cells in culture; top panel shows DAPI counterstaining of a nucleus from a cell immunopositive for the cytoskeletal and neural stem/progenitor cell marker proteins nestin (red) and GFAP (green). The panel below shows a single double labeled S-NSC cells double labeled for nestin and GFAP, merged image. The S-NSC double labeled cell interestingly possesses the same morphology and immunostaining pattern of cytoskeletal elements as originally shown in immunocompromised mouse xenografts of the original parental line following intracerebral transplantation and their homing to neurogenic regions (Fig. 3f in Walton et al. Development, 2006^49^). (**b**) Heat maps showing differentially **e**xpressed protein coding and miRNA genes. Left panel, L-NSC microarray gene expression data. Upregulated and downregulated genes in infected cells are shown in red and blue respectively. Middle panel, S-NSC differentially expressed protein-coding genes. Red and green represent genes over- or under-expressed in infected cells respectively. Right panel, microRNA genes over- (red) and under-expressed (green) in infected S-NSC. (**c**) Functional enrichment analysis of transcriptomics datasets focused on KEGG pathways and GO Biological Processes. Left panels, enrichment analysis on L-NSC transcriptomes; right panels, enrichment analysis on MM6 cells and Steindler’s NSC and NDC cells. Red arrows indicate interesting pathways. DEGs, GO Biological Processes enriched with DAVID software v6.7. GO Biological Processes, p-value < 0.01, number of genes associated with certain GO term >5. (**j**) KEGG pathway enrichment analysis. Identified DEGs, KEGG pathways enriched with DAVID software v6.7. KEGG pathways, p-value < 0.05. For L-NSC GO and KEGG analysis show neddylation, pathways of Alzheimer’s, Parkinson’s and Huntington’s diseases. For S-NSC there are a variety of interesting pathways marked by red arrows, as in MM6 and NDCs as well involving ribosomes, p53 signaling, cell cycle, TGFβ, purine metabolism, NOD receptor signaling, MAPK signaling, vesicle mediated transport among others. Nominal p values were utilized for KEGG and GO analyses; p-values for pathways that were robust to Benjamini Hochberg correction also are shown in Supplement B: Table S16, 17. Comparison of the nominal and corrected p values indicate the most robust pathways.

**Figure 4:**
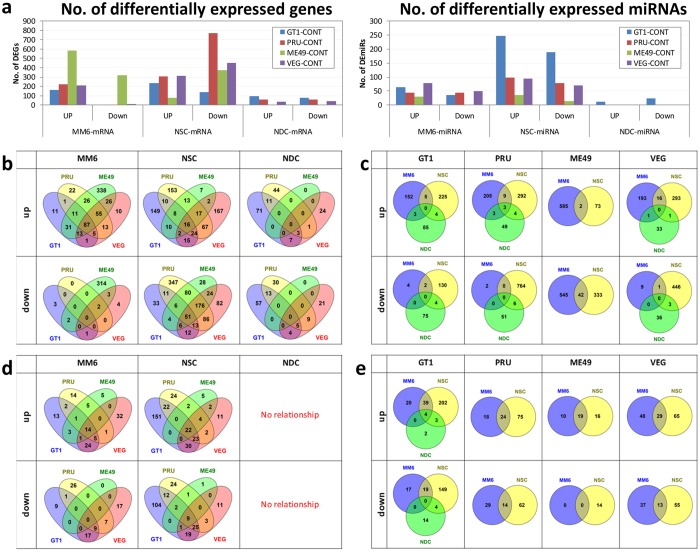
Comparative analysis of MM6, S-NSC and S-NDC transcriptomics profiles by cell type and parasite strains. (**a**) Number of protein-coding genes (DEGs, left panel) and miRs (DEmiRs, right panel) differentially expressed between infected and uninfected conditions. This is with a false discovery rate ≤0.01 and absolute log2-fold-change ≥1. (**b**–**e**). Number of shared over- or under-expressed protein-coding genes (**b** and **c**) or miR genes (**d** and **e**) grouped by host cell type (**b** and **d**) or parasite type (**c** and **e**). Both cell type and parasite strain drive differential response, with a predominant effect from the host cell type. Nominal p values were utilized for KEGG and GO analyses. Pathways that were robust to Benjamini Hochberg correction also are shown in Supplement B: Tables S16, 17.

Human primary neural stem/progenitor cells (stem/progenitor, stem or progenitor can be used to describe them because the degree of stemness of the two primary cell lines studied here is still not certain, even though both are highly potent clonogenic cells that should have many common phenotypic characteristics since both lines were isolated and expanded from human hippocampus following temporal lobectomy for intractable epilepsy^48, 49^) were selected for these studies because they are multipotent cells central to neurodevelopment and neuroplasticity. To determine whether *T. gondii* can perturb human neural pathways that contribute to the generation, differentiation and survival of normal circuitry components, including cell death and protein misfolding and degradation, we characterized the host cell functional response, at transcript and protein level, of these two primary human temporal-lobe neural stem cell lines (NSC, Table 2) generated in two different laboratories. One of these cell lines, we named L-NSC, is a partially differentiated neuronal stem/progenitor cell^48^. L-NSC (Fig. 3a left panel, Table S3) is known to immunostain with antibodies to well-established neural stem cell/astrocytic and neuronal progenitor markers Nestin and glial fibrillary acidic protein, “GFAP” (data not shown here). L-NSC are shown here to also be immunoreactive for NFκB which is involved in DNA transcription, cell survival, cytokine and growth factor production, and Stat3 which is also involved in cell growth and survival. These cells, as well as the second neuronal stem cell line studied named S-NSC, are able to take up the thymidine analogue bromodeoxyurdine, “BrdU”, indicating their ability to replicate and expand impressively *in vitro*. The second NSC studied was initially isolated and characterized by Steindler *et al*.^49^ (S-NSC, Supplement B: Table S3). These genetically distinct, primary human temporal-lobe neural stem/progenitor cells also express the neural stem cell markers Nestin and GFAP and can be differentiated into neurons and glia both *in vitro* and following intracerebral transplantation^49^ (Fig. 3a right panel, showing phase microscopic and immunostaining for astroglial stem/progenitor cell markers of the human neural progenitor cell line). S-NSC allowed us to study a second biologically distinct human brain NSC to compare with our L-NSC. Because of distinguishable isolation and *in vitro* growth conditions between the two laboratories, along with known interclonal heterogeneity that has previously been shown for clonogenic NSC lines even generated from the same human hippocampal specimen^50^, it is always anticipated that profiling of the two lines under any condition should reveal similar but not identical molecular signatures. For both cell types, and the differentiated form of S-NSC (S-NDC), the transcriptional responses were characterized using differing *in vitro* culture conditions (Table 2), with and without *T. gondii* infection. These studies were complemented with transcriptomics analyses on a human monocytic cell line (MonoMac6, MM6, Supplement B: Table S13)^25^ to reflect inflammatory cells that enter the human brain. All four types of cells were infected with parasite isolates representative of the three clonal types of *T. gondii* that predominate in the U.S. and Europe (Types I, II and III). To characterize the transcriptional response of each of the four human cell types to the infection of genetically diverse parasite strains, we performed differential gene expression analyses of both protein-coding (L-NSC, S-NSC, S-NDC and MM6) and miRNA-coding (S-NSC, S-NDC and MM6) genes for each infected culture condition compared to its respective uninfected control. Figures 3 and 4 summarize results of RNA profiling and analysis of the *T. gondii* RNA brain infectome for L-NSC, S-NSC, S-NDC and MM6 cells. Significant modulations (>1.5 log fold change of infected versus uninfected cells) were evident at 18 hours post infection with Type I, II and III *T. gondii* tachyzoites. The bar on the heat map for L-NSC is the RMA normalized intensity.

**Table 2:** Neuronal cells, culture conditions, and markers.

Cell	Medium	Serum	Growth Factors (ng/ml)			Other	Markers	Days to Differentiation
			EGF	bFGF	NGF			
NPC-LS*	DMEM/F12	0,5, or 10% FCS	20	20	0	N2 supplement/ 1 μg/ml laminin	Nestin, GFAP, Neur	N/A
*S-NSC*	DMEM/F12	5% FCS	40	40	0	N2 supplement/ Bovine Pituitary Extract	Not Present	0
*S-NSC-LNS*	DMEM/F12	0	0	0	25	c-AMP 05 um IBMX 0.5 uM	Rare: Nestin, GFAP, Neur	14
*S-NDC*	DMEM/F12	0	0	0	25	c-AMP 05 mM IBMX 0.5 mM	Nestin, GFAP, Neur	7

*Called L-NSC.

The relatedness of results from all four types of host cells and identification of the signature pathways that the parasites modulate were analyzed by heat maps (Fig. 3) and gene set enrichment analysis using Gene Ontologies (GO) and KEGG pathways knowledgebase (Fig. 3c, Supplement B: Tables S16 and S17). This comparison revealed critical similarities and differences between different cell types and culture conditions. Some of these modulations were unique to different parasite strains and cell types. The heat maps also showed effect of host cell type in Fig. 3b. The overlap and differences of expression are shown in Fig. 4a–e. In MM6, modulation of P53 signaling, JAK-STAT signaling, programmed cell death, arachidonic acid metabolism, response to hypoxia were noteworthy (red arrows in Fig. 3c, right panels). Effects on translational elongation, apoptosis, cell cycle, vesicle mediated transport, ribosomes, amino acid metabolism, TGFβ signaling, p53 signaling, MAP kinases, circadian rhythm, and cell cycle were some especially noteworthy in GO and KEGG pathways for S-NSC (red arrows in Fig. 3c). In infected L-NSC, transcriptomes also showed that parasites modulate pathways of sensory perception of smell, mitochondrial organization, protein modification by small protein conjugation, cognition, neurologic system processes, neddylation, oxidative phosphorylation, G protein coupled receptor protein signaling, androgen and estrogen metabolism, ribosomes, translational elongation, and particularly noteworthy, pathways of Parkinson’s, Alzheimer’s and Huntington’s diseases (Fig. 3c). Specific genes in the L-NSC pathway analysis are shown in Tables 16 (GO) and 17 (KEGG). Specific *T.gondii* susceptibility genes that also contribute to these pathways and functions are in Table 1 and Supplement B: Tables S16 and S17. This transcriptomic analysis also served as a basis for an analysis of upstream regulatory genes shown later.

It was notable that a significant number of pathways modulated by *T. gondii* in human NSC reflect immunologic mechanisms. This made the comparison of the transcriptomic differences between NSC and this monocytic cell line at a stage before maturation (Mono-Mac 6) of interest. This allowed us to identify the distinct and common modification in each of these cell types. We also compared their messenger RNAs (mRNA) and micro-RNAs (miRNA) with NSC that are stimulated (NDC) by Nerve Growth Factors for 7 days. Human MM6, NSC, and NDC each were infected with Type I (GT1), or Type II (PRU or ME49) or Type III (VEG) parasites for 18 hours. The differential transcriptional responses are significantly different between NSC and NDC, as well as when compared to MM6 (Fig. 4). Direct comparison of the transcriptome profiles also demonstrated that differences in protein and miRNA coding gene expression were mostly influenced by host cell types rather than parasite type, in particular for MM6 and S-NSC cells (Fig. 4a–e). This comparison further demonstrated the substantial numbers of human genes modulated during parasite infection that were not shared among the different host cell types assessed and the NDCs in different culture conditions. This comparison underscores immune-type responses in human neural stem cells but also that they are not the only pathways perturbed. In addition, we identified some interesting parasite-specific pathways perturbations. Notably, pathways for Alzheimer’s, Parkinson’s, and Huntington’s diseases have the same genes being affected by infection modifying oxidative phosphorylation that were prominently modulated for L-NSC, but only when infected by Type II (ME49) parasites. Baseline levels also differed between cell types (Fig. S1).

Significant changes in gene expression were also evident by miR-seq for MM6, S-NSC and S-NDC cells at 18 hours post-infection (Figs 3 and 4 and Supplement B: Tables S11 and S12). These included mirs 139, 132, 29a, 107, 218, 143, 155, 199a, 21, 16, 181b-1.

**Figure 5:**
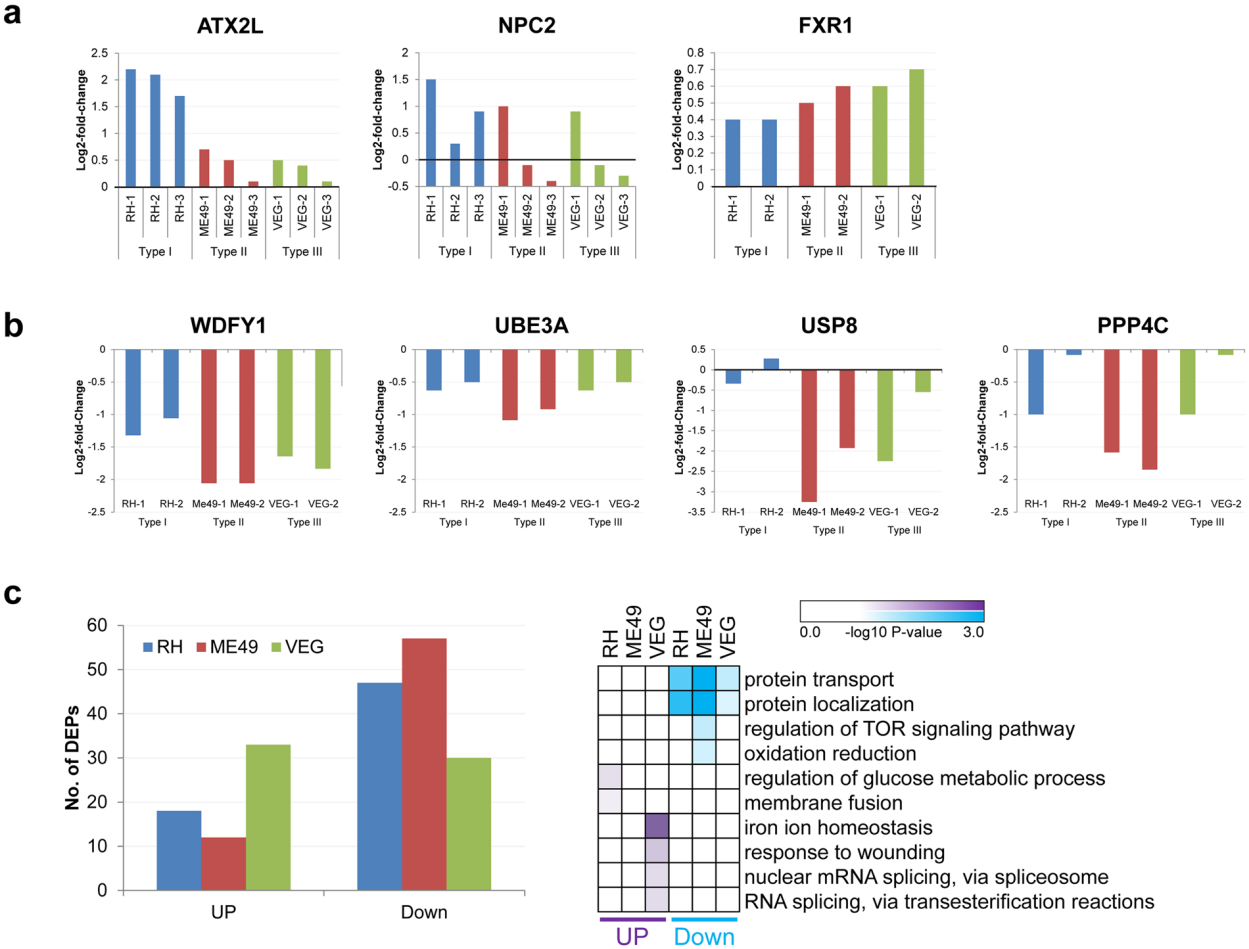
Proteomics and their Analyses. a-b. Proteins differentially expressed during parasite infection of L-NSC (**a**) or S-NSC (**b**). ATXN2L, Ataxin 2-like; NSC2, Niemann-Pick disease, type C2; FXR1, Fragile X Mental Retardation Autosomal Homolog 1; WDFY1, WD Repeat And FYVE Domain Containing 1; UBE3A, Ubiquitin Protein Ligase E3A; USP8, Ubiquitin Specific Peptidase 8; PPP4C, Protein Phosphatase 4 Catalytic Subunit). (**c**) Left panel, number of differentially expressed proteins (DEPs) in S-NSC infected with *T. gondii* types I, II and III; right panel, GO Biological Processes significantly overrepresented (p-value < 0.01) in the set of 3,359 proteins differentially expressed in infected S-NSC compared with their respective uninfected controls (>2-fold change and false discovery rate <0.1). Nominal p-values were utilized for KEGG and GO analyses. Pathways that were robust to Benjamini Hochberg correction also are shown in Supplement B: Table S15.

**Figure 6:**
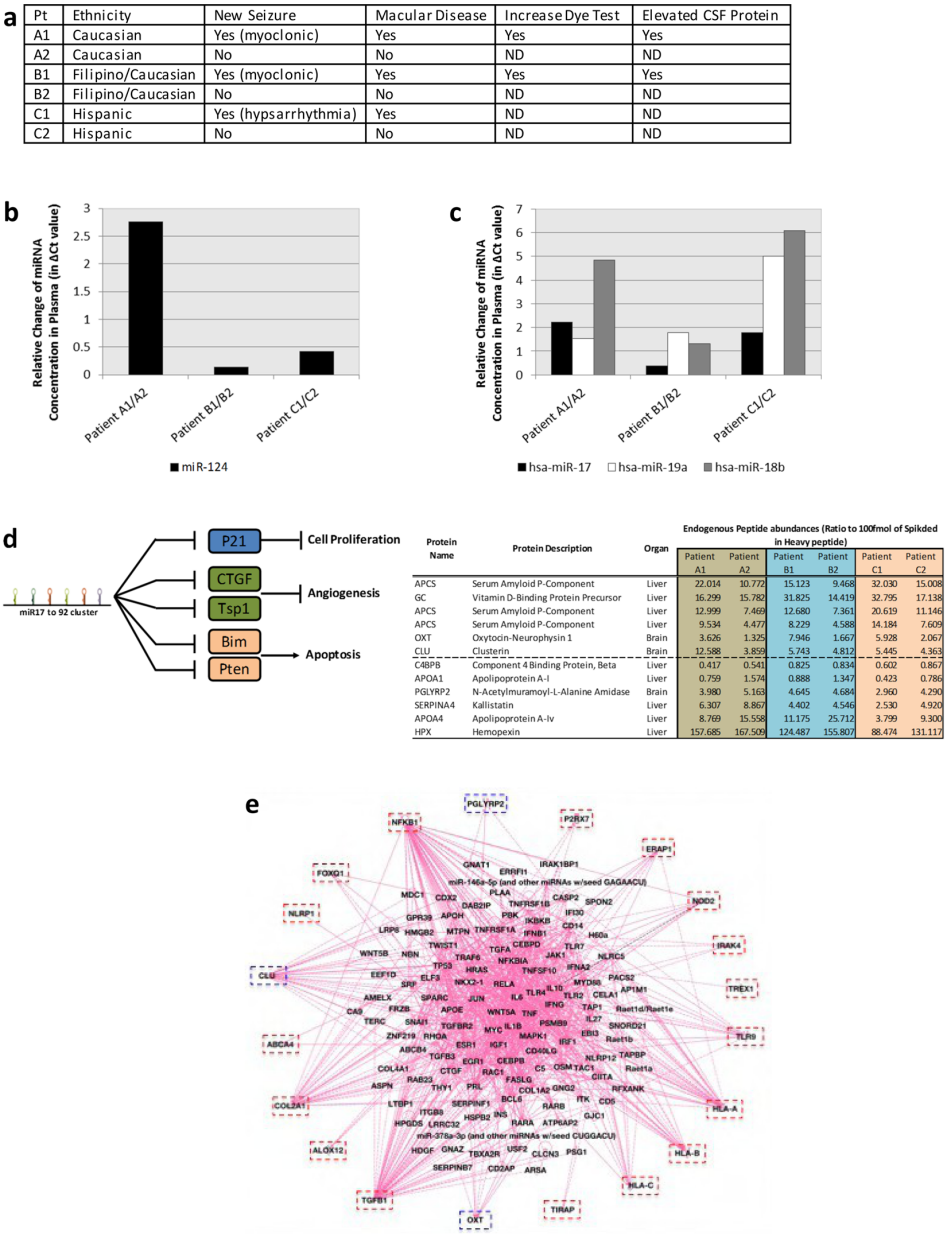
Serum biomarkers from boys with active brain disease due to *T. gondii* reflect infection and neurodegeneration. (**a**) Tabular clinical summary: Three pairs of children, matched demographically; one in each pair had severe disease and the other mild or no manifestations. One pair dizygotic, discordant twins. Each ill child had new myoclonic or hypsarrythmic seizures. Two children had T2 weighted abnormalities on brain MRIs similar to active inflammatory and parasitic disease in murine model^4^. (**b**–**d**) Protein and miRNA serum biomarkers: Panel of protein and miRNA profiling performed on serum obtained at time of new illness. Changes in serum miRNA concentration between each infected child and corresponding control is expressed as the difference in RT-qPCR Ct-values for miR-124 (**b**) and miR-17, miR-19a and miR-18b (**c**). Abundance of peptides measured. In Fig. 6b and c, these data are extracted directly from the qPCR panel for miRNA profiling. (**d**) Left panel, schematic representation of the genes targeted and pathways modulated by miRNA clusters 17–92; right panel, peptide abundances from the 10 most intense peptide ions detected by proteomics in the three children pairs. Peptides with higher or lower abundance in ill children compared to healthy controls are depicted above or below the dashed line respectively. (**e**) Bundling of upstream regulators predicted from susceptibility genes (red box) and brain biomarkers (blue box). See Supplement 2 for IPA analysis of upstream regulators with p-value <5 × 10^–3^. Circulating biomarkers detected in the *T. gondii* infected brain are clusterin (CLU), oxytocin/neurophysin I prepropeptide (OXT), peptidoglycan recognition protein 2 (PGLYRP2), and microRNAs (miR214, miR-17, miR-18b, miR-19a) (Fig. 4B, Supplement B: Table S7). These specific miRNAs were not annotated in the IPA database, so the analysis focuses on the 3 protein biomarkers. PGLYRP2 is a hydrolase that recognizes and digests bacterial active peptidoglycan into biologically inactive fragments that triggers innate immune responses to intracellular pathogens. Clusterin/Apolipoprotein J is a secreted chaperone which is proposed to be a biosensor of oxidative injury. The ‘love/bonding hormone’ Oxytocin is a posterior pituitary hormone that is synthesized in the hypothalamus. OXT hormonal activity influences cognition, tolerance, adaptation and complex sexual and maternal behavior, as well as the regulation of water excretion and cardiovascular functions. Presence of markers of neurodegeneration and inflammation include Clusterin, PGLYRP2, and Oxytocin in ill children compared with their healthy controls.

**Figure 7:**
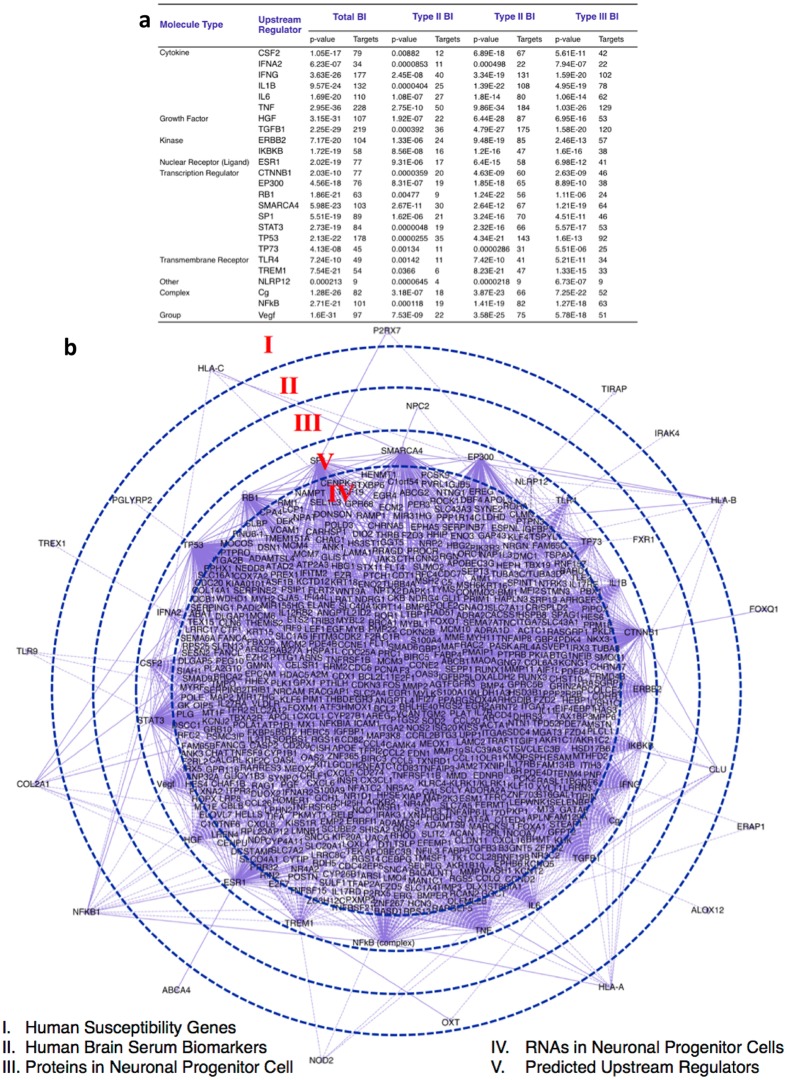
Deconvolution of total brain infectome reveals upstream regulatory pathways. **a.** Statistical probability of 25 upstream regulators of total brain infectome (BI) with most significant p-values. The Total Brain infectome included 1,678 genes from all datasets of genetics susceptibility, brain biomarkers, messenger RNAs of L-NSC and S-NSC, and proteins of L-NSC. The BI is segregated into Type I, II and III infection (see Supplement B: Table S7). IPA analysis of BI identified 1,640 upstream candidates (see Supplement B: Table S7). ‘Target’ indicates the number of *T. gondii*-induced genes found in each upstream regulatory pathway. TNF, TGFβ1, IFNG, TP53 and IL1β are the most dominant upstream regulators found in the *T. gondii* brain infectome. (**b**) “Orbital” visualization. The 25 highest statistical valued upstream regulators (**a**) are added to the brain infectome and graphically mapped by IPA. The relationships between the 5 reconstruction layers are visualized in the “orbital diagram”. Specific genes are manually repositioned in each empirical ‘layer’ drawn to ‘orbit’ the core gene datasets. Upstream regulatory network (V) connects RNA (IV) and Protein (III) of human NSCs, brain biomarkers (II), and NCCCTS Susceptibility genes (I). Since the IPA drawing program is limited to 30,000 interactions lower than the cut-offs, ATXN2L was not found in this visual analysis. The total brain infectome is deconvolved into functional correlates by IPA Core Analysis. Canonical pathway annotation reveals the predominant mechanism of IL-17 pathways in arthritis, psoriasis and allergy with related cell types (macrophages, fibroblasts, endothelial cells, osteoblasts, osteoclasts, chondrocytes) playing a role in the inflammation. Top pathways also include cardiogenesis, adipogenesis, hepatic stellate cell activation and diapedesis of agranulocytes and granulocytes, molecular mechanisms of cancer accompanied with the signaling of colorectal cancer metastasis. Wnt/Ca + pathway moderates axonal guidance signaling and other cell growth and developmental pathways. The top scored canonical pathway shows also cell cycle control of chromosomal replication as possibly a prevailing molecular mechanism. Supplement B:Table S8 describes the IPA analysis of canonical pathways with functional mechanisms in neural stemness, neurodevelopment, neurobiology, immunology, cancer, and cell cycle. Supplement B: Table S9 identifies pathways associated with the nuclear factor NFkB.

L-NSC and S-NSC infections with Type I, II, III parasites were also studied using iTRAQ quantitative proteomics (Fig. 5). Differential protein expression analysis of L-NSC cells identified only three primary candidate proteins with significant fold change increases in infected cells. These proteins were Ataxin 2-like protein (ATXN2L); Fragile X-related protein 1 (FXR1); and Niemann-Pick-like protein (NPC2) (Fig. 5a, Supplement B: Table S4). An IPA later showed how these three proteins were linked to transcriptomic infectomes and identified congenital toxoplasmosis susceptibility genes (Figs 6, 7). These proteomics and transcriptomics (Figs 3–5) defined empirically, responses of human L-NSC to infection with *T. gondii*.

Quantitative proteomics of S-NSC identified 20 proteins that presented at least a significant (p < 0.05) 1.5-fold change in abundance in response to *T. gondii* infection compared to uninfected controls (Supplement B Table S15). Interestingly, this includes a number of proteins that participate in the regulation of the immune response, cell proliferation and cancer, protein ubiquitination, and neurodevelopmental disorders. Modulated proteins of interest included WDF41, PPP4C, USP8, UBE3A, ECF1A2 (Fig. 5b, Supplement B: Table S6). Contrary to L-NSC, S-NSC cells did not show any significant change in the level of ATXN2L, FXR1 or NPC2 proteins. S-NSC proteomics also revealed that some of the pathways whose gene expression was perturbed during *T. gondii* infection (Fig. 5b,c) were also modulated at the protein level. (Please see response to wounding and regulation of TOR-signaling pathway in Fig. 5c, right panel; Supplement B Table S15). In summary, S-NSC proteomics, pathway and GO enrichment analyses showed signaling pathways impacting on cell death, TOR, protein transport/localization and RNA splicing. Oxidation reduction, vesicle mediated transport, iron homeostasis and glucose metabolism were also influenced, some differentially by parasite type (Fig. 5c). Consistent with these enrichment results, prediction of alternative spliced isoforms in our transcriptomics datasets identified a number of candidate genes predicted^51^ to be differentially spliced during parasite infections (Supplement B: Table S14).

#### Reconstruction 3: Serologic biomarkers reflect infection and neuron damage

Human serum biomarkers of three ill congenitally infected children from the NCCCTS (A1,B1,C1 in Fig. 6a–c) reflected *T. gondii* infection and neuronal damage when compared against age, sex, and demographically matched controls (A2,B2,C2) in Fig. 6a–c. Each of the three ill children had new myoclonic-“infantile” spasms, or hypsarrhythmic seizures (Fig. 6a). For two of them, A1 and B1, this was associated with a rise in or high *T. gondii* specific IgG antibody titers. IgG was not measured for the third ill child, C1. Child C1, who decades earlier, was recognized to have congenital toxoplasmosis and later developed hypsarrhythmic seizures decades. A1 and B1, diagnosed more recently, had T2 weighted abnormalities in brain MRIs. C1, with this clinical problem was cared for prior to the development of regular clinical use of MRIs. The findings in the ill children were similar to active inflammatory and parasitic caused brain disease such as epilepsy and motor abnormalities, and the T2 weighted abnormalities, seen in a murine model^4^. These similarities prompted us to look for serum biomarkers for active brain damage in the setting of chronic congenital toxoplasmosis. In the murine model studies, T2 weighted abnormalities in MRIs correlated with chronic and active inflammatory brain pathology characterized with light and electron microscopy studies. To identify potential serologic biomarker signatures associated with the aforementioned neurologic manifestations of toxoplasmosis, we assessed a panel of 700 miRNA transcripts by RT-qPCR and performed proteomics on serum samples obtained at the time of appearance of this new illness for the ill children and at the same age for the control children. These miRNA panels had shown biomarkers associated with neurodegenerative diseases^52^. Serum miRNA profiling detected four miRNA transcripts with more than 2–fold increases in the sera of at least one of the three infected children compared to their paired healthy controls (Fig. 6b and c). These include mirs 17, 186, 19a, 124 all encoded by miR 17-92 family cluster. Proteins that increased included clusterin (CLU), serum amyloid P component (APCS), and Oxytocin (OXT) (Fig. 6d,e). N-acetylmuramoyl-L alanine amidase (PGLYRP2) and Apolipoprotein A1 (APOA1) decreased (Fig. 6).

To elucidate potential relationships between candidate serologic biomarkers, our susceptibility genes and their upstream regulatory pathways, and toxoplasmosis, we performed IPA network analysis (Fig. 6e). This analysis revealed that biomarkers from sera of some cohort members, and susceptibility genes and their upstream regulators are interconnected. IPA analysis was performed to elucidate potential relationships between candidate serologic proteins and toxoplasmosis susceptibility genes, such as upstream regulators (Fig. 6e, Supplement B: Tables S2 and 7). Comparison of top-scored IPA canonical analysis showed that atherosclerosis signaling may relate to interactions between CLU and proteins encoded by congenital toxoplasmosis susceptibility genes (*TGFβ1*, *COL2A1*, *ALOX12*, *NFκB1*). Atherosclerosis was also facilitated by association with other genes in RNA infectomes of L-NSC (2 genes) and S-NSC (10 genes). Disease and function analysis indicated that additive effect of congenital toxoplasmosis biomarkers activate mechanisms of immune cells and neurons, synthesis of prostaglandins and a dominant shift to cancer potentiation.

#### Deconvolution 1. IPA analyses reveal upstream regulators

First using only the findings from L-NSC, the four primary original data sets were combined as a total brain infectome. The sum is 1,678 human genes that were assembled from Type I (347 genes), Type II (1,225 genes), and Type III (801 genes) parasitic infections (Supplement Table S7). We determined by IPA analysis the top 25 upstream regulators and created an ‘Orbital’ design to visualize the connections between 5 “layers” (Fig. 7). Each data set represented a ‘layer” visually: (I) Human susceptibility genes; (II) Human brain serum biomarkers; (III) Differential proteins in neuronal progenitor cell (L-NSC); (IV) Differential RNAs in neuronal progenitor cells (L-NSC, S-NSC); and (V) Predicted Upstream Regulators.

The top-ranked 25 upstream regulators were modulators of important pathways of the neural, immune and endocrine systems, including cytokines (CSF2, IFNA2, IFNG, IL1B, TNF), growth factors (HGF, TGFβ1, VEGF), immunological transmembrane receptors (TLR4, TREM1), transcriptional regulators (CTNNB1, EP300, RB1, SMARCA4, SP1, STAT3, TP53, TP73) and hormone regulators (ESR1, Cg complex) (Fig. 7). Predicted upstream regulators connected the proteins detected in infected NSC in layer III such as FXR1 to oncogene TP73 and NPC2 to SMARCA4. The brain biomarker (called “layer II”) also showed connectivity, e.g., hormone oxytocin detected in the infected brain serum associated with immunological upstream regulators TREM1 and IL6, whereas peptidoglycan recognition PGLYRP2 connected with oncogene TP53 regulatory networks. Portions of analyses are shown in Supplement B: Table S7.

**Figure 8:**
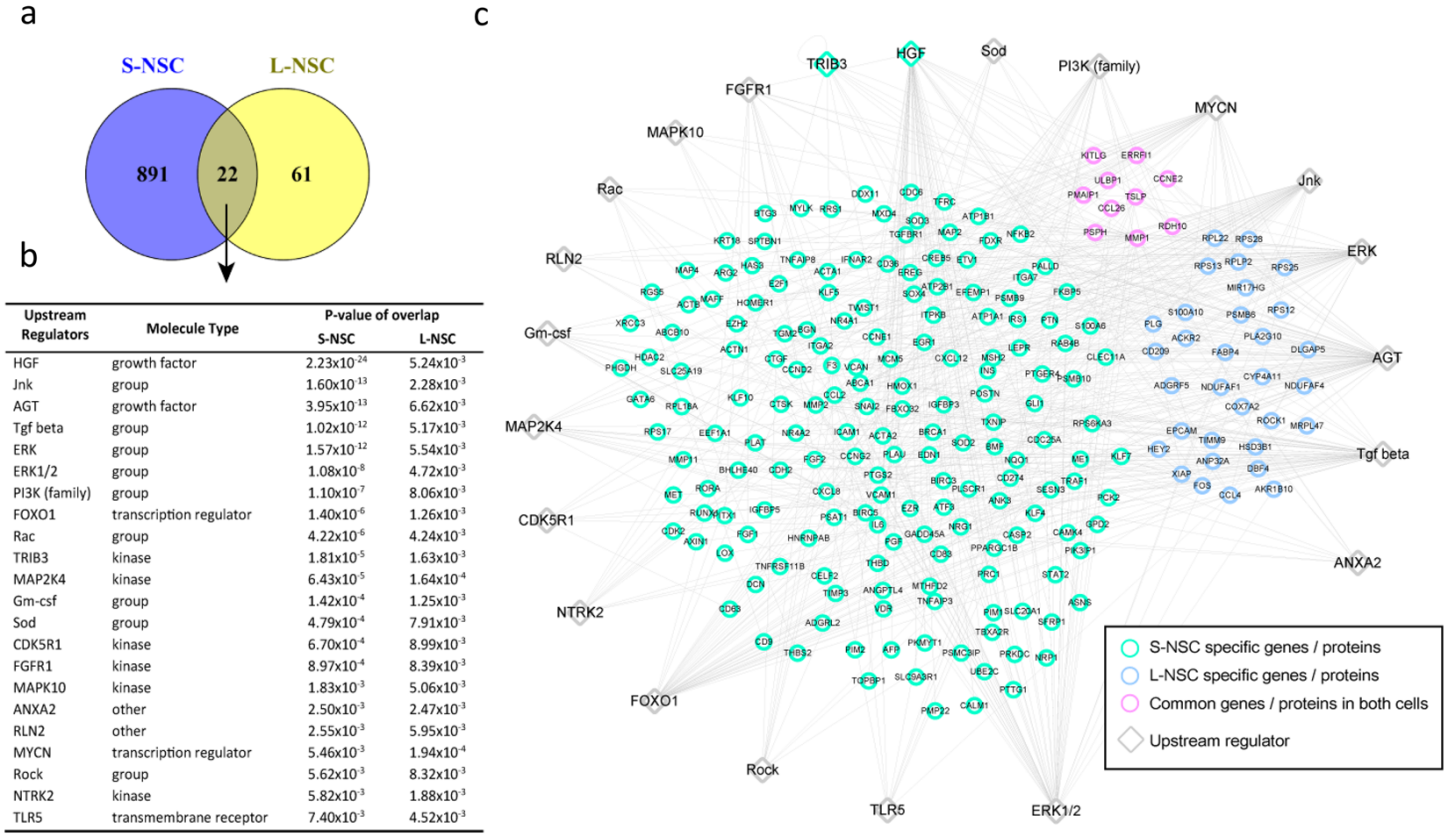
Upstream regulators targeting genes and proteins differentially expressed in S-NSC or L-NSC. (**a**) Relationships of upstream regulators: 913 and 83 molecules were identified as upstream regulators of genes or proteins differentially expressed in S-NSC and L-NSC, respectively (p-value ≤ 0.01). (**b**) Regulators in common. Venn diagram shows that among the upstream regulators, 22 molecules are common between L-NSC and S-NSC. (**c**) Gene regulatory network targeted by the 22 common upstream regulators.

The transcriptome and proteome of the S-NSC were not included in this initial analysis. Therefore, we performed an upstream regulatory gene analysis (Fig. 8) which shows separate and overlapping upstream regulatory genes for our two experimental types of primary neuronal stem cells. This analysis includes all the genes identified by both transcriptomics and proteomics from L-NSC and S-NSC. The upstream regulators, had been shown earlier to be related to the susceptibility genes and circulating biomarkers in the ill children (Figs 2b, 7a,b). These are apparent and those upstream regulators that are common to both types of cells are seen in the Venn diagram and Table in Fig. 8. That the different cell types also have distinct upstream regulators is also notable in the Venn diagram (Fig. 8). The colors in Fig. 8 indicate which analysis and type of cells contribute which components to this upstream regulator analysis. Some of the genes critical in a variety of chronic complex disease pathways, immune response, and neurodegeneration are seen in this analysis. A few examples of key upstream regulators and their functions are *NFkB, VEGF, MYC, EGFR, FGF, TGFβ, PI3K/AKT/PTEN, ERK, PI3K, FoxOs, GM-CSF, and FGRR1*. Additional details of and from this analysis is provided in the methods.

#### Deconvolution 2. Cluster protein interactions reveal critical mechanisms of pathogenesis

**Figure 9:**
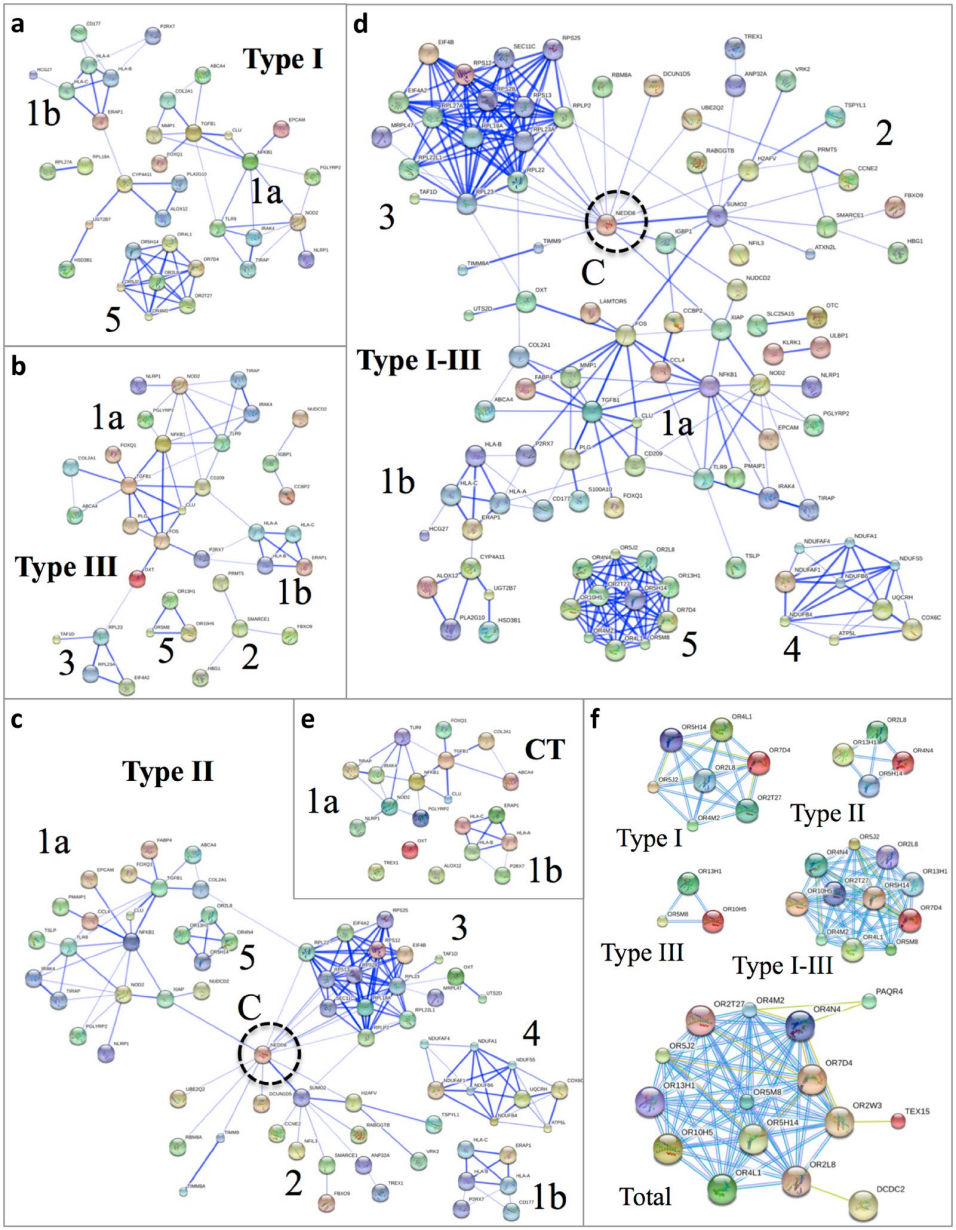
Cluster deconvolution uncovers six clusters of protein-protein interactions effecting brain functions and circuitry. STRING^192^ analysis of the brain infectome was carried out to elucidate protein-protein interaction networks modulated by parasite infection (see Supplement B: Table S6). STRING analysis was performed on a dataset composed of human susceptibility genes and biomarkers identified from patients with congenital toxoplasmosis (CT, panel e) or plus a collection of genes differentially expressed in L-NSC infected with type I parasites (panel a), type III (panel b), type II (panel c) or all strains (panel d). Six distinct clusters were visualized from the integration of genetics, biomarkers and L-NSC expression data (panel e) in which clusters 1–3 radiate from NEDD8 (dashed circle, panel c,d), a central node modulated during Type II infection (panel c). The genetics and biomarkers generated clusters 1a and 1b that were further expanded with connections to genes modulated in Type I-III infectomes (panel a–c). Clusters 4 and 5 do not interact with NEDD8 (panel c and d). Panel f shows a detail of the genes belonging to the odorant receptor cluster 5 that are perturbed in L-NSC infected with *T. gondii* type I (Type I), II (Type II), III (Type III) or all three types (Type I-III) plus the sum of genes modulated in S-NSC infected with types I, II and III parasites (Total). In panels a-e edge thickness indicates confidence of interactions, with thin edges having middle confidence combined scores and thick edges high confidence combined scores, as defined by STRING. In panel f, edge color represent interaction evidence source as defined in STRING: light blue, curated database; yellow, text mining; purple, protein homology. Note: odor attraction of mice and chimpanzees to cats^56, 126^.

Our second deconvolution approach used network analyses of protein-protein interactions on L-NSC total infectome (Fig. 9). Whereas IPA is designed to annotate pathways and networks, STRING database and web tool provide an analysis that detects protein-protein interactions^53^. Due to the limit of 30,000 interactions in STRING graphical tool, clustering deconvolution was only completed for L-NSC brain infectome (susceptible genes + biomarkers + L-NSC RNA + L-NSC proteins). We analyzed separate and combined data sets of Type I, II, and III infections.

*T. gondii* brain infection was deconvoluted into six simplified functional clusters (Fig. 9, Supplement B: Table S6). Four clusters (1a, 1b, 2, 3) pivoted around a central node defined by NEDD8 (neural precursor cell expressed, developmentally down-regulated 8). This central node was potentially involved in hijacking cellular proliferation and cell death by either protein neddylation or sumoylation. The largest Cluster 1a was predominantly associated with cellular movement and migration that utilizes mechanisms described for immune and endocrine signaling. Cluster 1b appeared to be more specific for leukocyte migration and may involve parasitism of brain’s lipid metabolism. Cluster 2 also mediated cellular proliferation but appears to be more specific to the role of ubiquitin-mediated protein degradation in cell cycle control. Cluster 3 pinpointed the parasite’s hijacking of protein synthesis to regulate the host cell cycle. Cluster 4 revealed the parasite’s capacity to modulate the brain’s ATP production by mitochondrial oxidative phosphorylation. Cluster 5 suggested the alteration of olfactory response due to brain parasitism. Nedd8 is a critical, key central node (Fig. 9).

#### Deconvolution 3. *T. gondii* modulates proteins involved in neurodegeneration, epilepsy, motor diseases and malignancy

**Figure 10:**
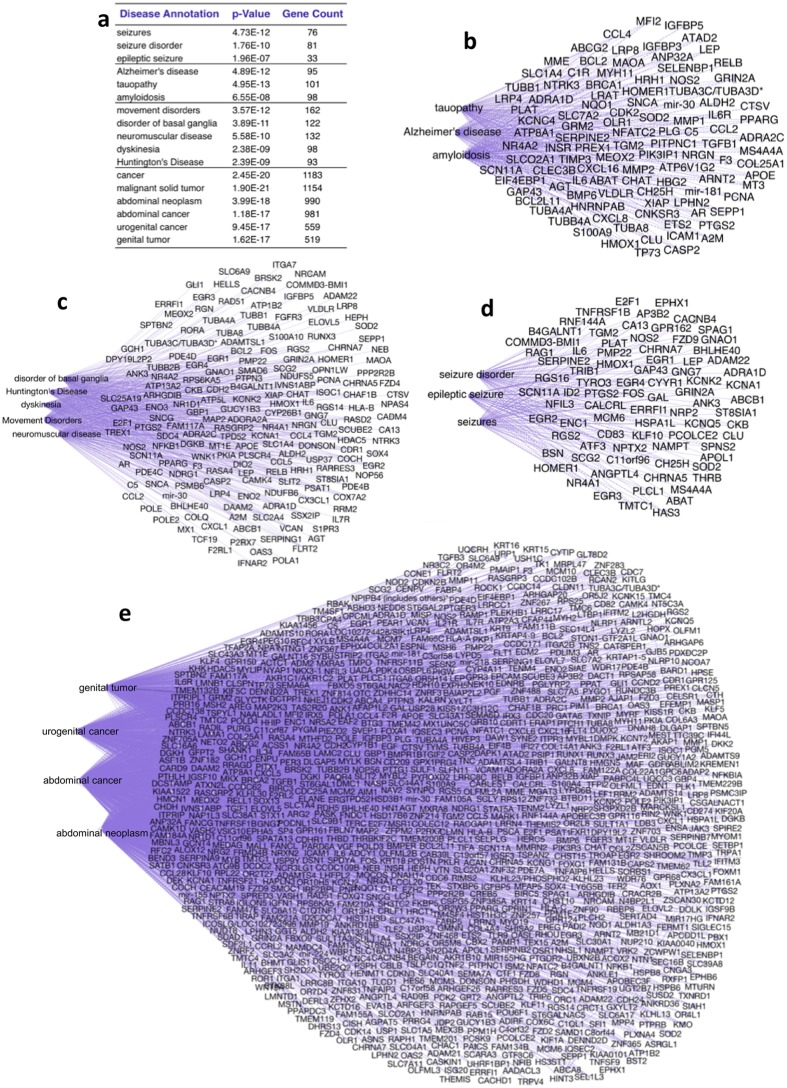
Deconvolution of brain infectome by disease correlation. The 3rd deconvolution approach of the total brain infectome predicts correlation between *T. gondii* infection and common neurological diseases using IPA disease and function tool (see Supplement B: Table S10A,B). (**a**) Grouping of disease annotations included p-value and group size. The 4 disease groups are detected for all Type I, II and III infections. (**b**) Alzheimer group included Alzheimer’s disease, tauopathy and amyloidosis annotations. IPA Canonical analysis indicates this disease gene network is mediated by signaling pathways of adaptor protein 14-3-3 (7 genes), retinoic acid receptor LXR/RXR (7 genes), tumor suppressor protein p53 (7 genes), cytokine IL-17A (5 genes) and glucocorticoid receptor (13 genes). (**c**) Movement disorders. This entails predictions for movement disorders, disorder of basal ganglia, neuromuscular disease, dyskinesia and Huntington’s Disease. IPA canonical analysis of this gene network indicates signaling pathways mediated by G-Protein coupled receptor (13 genes), by cAMP (10 genes), transcriptional regulator 14-3-3 (7 genes), cytokine Endothelin-1 (8 genes), polypeptide hormone relaxin (9 genes) and corticotropin releasing hormone (8 genes). (**d**) Epileptic disorders pathways for seizures, seizure disorder and epileptic seizure. IPA canonical analysis of the 81-gene network indentifies top-ranking signaling pathways that are associated with retinoic acid receptor (6 genes), endothelin-1 (6 genes), Gaq protein (5 genes) and corticotropin releasing hormone (5 genes). (**e**) Cancer group. This illustrated mechanisms of cancer, malignant solid tumor, abdominal neoplasm, abdominal cancer, urogenital cancer and genital tumor that are potentially activated in the infected brain. The top 10 canonical pathways (Supplement B: Tables S8,9) reveal important mechanisms that may potentiate cancer development in the *T. gondii* infected brain, such as Wnt/Ca + pathway (14 genes) and role of IL-17A in arthritis (14 genes). Wnt/Ca + pathway annotation contains 6 frizzled class receptors (FZD2, FZD3, FZD4, FZD5, FZD8, FZD9) and receptor tyrosine kinase-like ROR1. Not shown in diagram is the association with the schizophrenia 19-gene network: ABAT, ABCB1, ADRA1D, ANK3, APOL1, CHRNA5, CHRNA7, E2F1, EGR3, EGR4, GAP43, GRIN2A, HOMER1, IL6, PMP22, PTGS2, SCG2, SOD2, TMTC1.

The third deconvolution approach was to predict global correlation between *T. gondii* infection and common neurological diseases using IPA disease and function tools. Only top-ranked canonical, diseases-functions and network pathways were included, with complete tabulations in Fig. 10. We discovered correlations with four major groups of epilepsy (81 genes), neurodegeneration (101 genes), motor disease (162 genes), and brain malignancy (1,188 genes) (Fig. 10, Supplement B: S10A,B). We found only 1 association with a schizophrenia network (p = 8.63E-12) of 19 genes.

IPA canonical analysis was performed to determine potential networks associating with diseases. Epilepsy gene group indentified top-ranking signaling pathways that were associated with retinoic acid receptor (6 genes), endothelin-1 (6 genes), Gq alpha protein (5 genes) and corticotropin releasing hormone (5 genes). Movement disorders mediated by the brain temporal lobe indicates signaling pathways mediated by G-Protein coupled receptor (13 genes), cAMP (10 genes), transcriptional regulator 14-3-3 (7 genes), cytokine Endothelin-1 (8 genes), polypeptide hormone relaxin (9 genes) and corticotropin releasing hormone (8 genes). The neurodegenerative disease gene network was potentially mediated by signaling pathways of adaptor protein 14-3-3 (7 genes), retinoic acid receptor LXR/RXR (7 genes), tumor suppressor protein p53 (7 genes), cytokine IL-17A (5 genes) and glucocorticoid receptor (13 genes). The most significant disease correlation to total brain infectome was cancer in which 1,188 of 1,678 genes are associated with a wide range of cancers (Fig. 7e, Supplement B: Tables S8; 10A,B). The top 10 canonical pathways determined by IPA reveal important mechanisms that may potentiate cancer development in the *T. gondii-*infected brain, such as Wnt/Ca + (14 genes) and inflammatory IL-17A (14 genes) pathways.

### Selected phenotypes*: T. gondii* type I, II and III induce activation of the NFκB pathway in human neuronal stem cells

**Figure 11:**
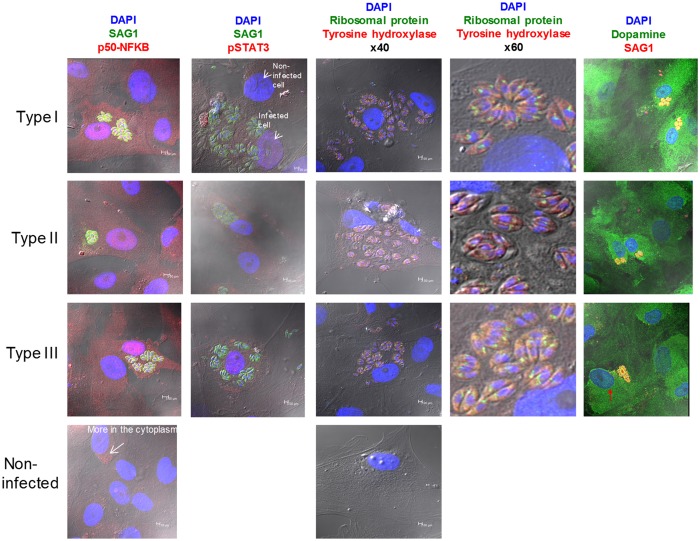
Phenotypes in NSC demonstrating functions that are biologically important empirically. NFkB (left panel): *T. gondii* (I, II, III) infection of S-NSC alters localization of p50-NFkB(red) and Stat 3 (second panel, red): SAG1 (Green), Hoechst (blue); *T. gondii*, in NSC, expresses or alters host cells’ neurotransmitters. Tyrosine Hydroxylase (red) in the infected NSCs that synthesizes dopamine is present in *T. gondii* (middle panels 40X, 60X). This is further exemplified in the furthest right panel by a dopamine-like immunostaining pattern in the parasite (green). The red arrow in the dopamine-like staining image points to a host cell dense perinuclear distribution of label. This suggests potential to influence neurotransmission in human NSC. This could contribute to abnormal circuitry function as seen in mice and as occurs in epilepsy in some persons^5, 6^. These experiments for immunostaining each of these molecules were performed at separate times, not simultaneously.

Reconstruction and deconvolution of the *T. gondii* infected brain provides a substantial number of biologically relevant mechanisms to investigate. A few selected phenotypes found in the informatics in our human neuronal stem cells were selected for study. These studies provide empirical validation of the conclusions from bioinformatics analyses (Fig. 11). Pathways identified through systems analyses of genetics, transcriptomics, proteomics, and with circulating biomarkers from matched pairs of well and ill children suggested that we might find empirically that these parasites would influence similar functional phenotypes in primary, human, neuronal stem cells which have not been characterized with Types I, II, III isolates of this common brain pathogen before. As downstream signaling molecules associated with an inflammatory process including NFκB and STAT3 had been identified in human neuronal stem cells (Fig. 2), we investigated whether Type I, II or III tachyzoites would modulate host gene expression in NSC by determining whether there was modulation of the NFκB pathway by the parasite. We infected S-NSC with Type I, II or III tachyzoites for 24 hours and followed translocation of NFκB p50 subunit and STAT 3 by immunofluorescence. As shown in Fig. 11, NFκB and STAT3 also were found in human neuronal stem cells in this experiment (Fig. 11a). We found that Types I,II,III tachyzoites infection of cells all affected nuclear localization of NFκB1 and STAT3 (Fig. 11a). Supplement B Table S9 shows canonical pathways that include NFκB1 determined by IPA.

Another selected phenotype studied was neurotransmitters in *T. gondii* and NSC. *T. gondii*’s effect on pathways associated with neurotransmission has been reported by others including effects on tyrosine hydroxylase, dopamine, and GABA receptors and distribution of GAD67 in murine brain infected by Type II but not Type III tachyzoites. To investigate whether either tyrosine hydroxylase or dopamine, is expressed by *T. gondii* tachyzoites when they are within NSC, we infected NSC with Type I, II, and III *T. gondii* tachyzoites for 24 hours. Type I, II, and III *T*. gondii tachyzoites in NSC immunostain with antibodies to tyrosine hydroxylase and dopamine. Both human host NSC dopamine and tyrosine hydroxylase were expressed in the parasite cytoplasm (Fig. 11b). We find this dopamine-like immunostaining in Type I, II, and III tachyzoites (Fig. 11b,c). We have not confirmed the exact nature and release of dopamine or other neurotransmitters including glutamate from these cells. A preliminary study (data not shown) also showed alterations of GABA and glutamate^5^ in S-NSC due to *T. gondii* infection (Roberts, El Bisatti, Zhou, McLeod *et al*., unpublished studies in progress).

### Intersecting susceptibilities and human diseases

Protein degradation and cell cycle are affected by *T. gondii*. Our transcriptomic and proteomic analyses indicated that this parasite alters neddylation (NEDD8) and ubiquitination which are key in clearing misfolded proteins, neuronal cell viability, and synaptic plasticity (Figs 3, 4, 9 and 11c). In the proteomics analyses, we also found that *T. gondii* modified alternative splicing (Fig. 5) and that an rMATS analysis^51^ predicted transcripts that are alternatively spliced based on the S-NSC transcriptomics data (Table S14) providing mechanisms whereby *T. gondii* may contribute to neurodegeneration, motor abnormalities, epilepsy, and malignancies (Figs 10, 11).

## Discussion

The Venn diagram in Fig. 1a provides a model and an explanation that addresses why some persons might have diseases to which *T. gondii* contributes, as suggested by our current analyses, and why other persons do not have these diseases. We hypothesize that disease occurs in the presence of the relevant susceptibility genes, parasite genotype and other innate and environmental factors such as other infections, the microbiome, or stress that influence immune responses, as shown in Fig. 1a. This reconstruction and deconvolution analysis presented herein (Fig. 1b,c) and summarized schematically in Fig. 1d unveils a plethora of pathways known in neurologic, immune and endocrine systems. These are likely to interact to cause neuropathologic diseases, depending on genetics of infected individuals (Figs 1a, 2a). Focus on neuronal stem cells reveals pathologic mechanisms in neurodevelopment and neuroplasticity. For example, cytokines mediate migration and homing of immune cells that serve parallel roles in spatial guidance of NSC in proper development and plasticity of human brain^54^. Cytokines stimulating CD8^+^ T cells to control *T. gondii* in brain^55^ may disrupt normal brain development and plasticity. A wide range of cytokines are evident in our data and interactome. Little is known about endocrine-type mechanisms in *T. gondii* infection^56^. This study provides a foundation to delineate endocrine influences and cross-talk with immune and neural systems.

Certain of our observations of specific genes/molecules, interactions, and pathways apparent in our data sets are particularly noteworthy: For example, in our genetics data (Table 1, Fig. 2), *TREX1, TLR9, TIRAP Mal, ALOX12, NALP1, NFκB*, and *TGFβ* connect to pathogen sensing, changes in lipids, and cell death and replication (Fig. 2). Characterization of phenotypes of our newly identified susceptibility genes is ongoing (Table 1): *TREX1* was studied because of the similarity between the brain calcifications in congenital toxoplasmosis and in the genetic Aicardi Goutiere’s disease that could be due to mutations in *TREX1* (Naranjo-Galvis *et al*., manuscript in preparation*). TIRAP MAL* (Hargrave *et al*., manuscript in preparation) (Table 1) was selected as a candidate gene studied individually as a downstream signaling molecule from TLR2 and 4*. FOXQ* (Table 1) was next chosen as a candidate gene because of a hydrocephalus association and subsequently has been shown to have a natural killer (NK) cell mediated phenotype^33^. *TLR9* was studied next as a candidate gene involved in recognition of small fragments of DNA (Hargrave *et al*., manuscript in preparation). Noting this association for *TLR9* in the NCCCTS (Table 1), we also have replicated the importance of *TLR9* in susceptibility to ocular toxoplasmosis in a cohort in Brazil^32^.

The localization of these *T. gondii* susceptibility genes in human brain (Fig. 2a) also provides insights consistent with our clinical observations of toxoplasmosis in mice and humans who develop seizures originating in the hippocampus-temporal lobe, impaired movement and hydrocephalus^4, 41^. Z-scores indicated that all of the 17 genes except *COL2A* are downregulated in the hippocampus. Memory, spatial navigation and control of attention occur in the hippocampus, which is also the dominant niche for neural stem cells. In the choroid plexus of the lateral ventricle where cerebrospinal fluid is produced, eight susceptibility genes are up-regulated (*ABCA4, HLA-A, HLA-B, HLA-C, IRAK4, NFκB1, TGFβ1, TREX1*), while five are down-modulated (*ALOX12, COL2A1, P2RX7, TIRAPMAL, TLR9*). In the globus pallidus that regulates voluntary movement, the susceptibility genes, other than *ABCA4, ALOX12, COL2A1, NOD2, TIRAP, TLR9*, are upregulated.

Upstream regulator analysis using IPA elucidates statistically significant upstream regulators including molecules which alter expression of downstream molecules. The systems analysis of upstream regulators of our susceptibility genes (Fig. 2b) underscores how this intracellular parasite influences the intricate balance between growth and death of its host’s cells. Upstream regulatory networks associated with these genes by IPA (Fig. 2b) include human genes that participate in inflammation, cell death, and cytokine signaling, as well as genetic, neurologic, and retinal diseases.

The transcriptomics analyses demonstrate some effects that are similar and some that differ between cell types (Figs 3 and 4). Possible explanations of the differences between cells include a number of variables including differences in the genetics of the host cells, fundamental differences in the cell types’ basal transcriptomics(Fig. S1), differences in culture conditions including media, growth factors and timing when cells were studied, or different responses to parasites. We also noted differences in responses when we studied another parasite called EGS which grows as encysted bradyzoites in tissue culture in HFF, MM6, and S-NSCs. Pathways that are perturbed suggest profound effects on host cells. These include for S-NSC: translational elongation, apoptosis, cell cycle, vesicle mediated transport, ribosomes, amino acid metabolism, TGF-β signaling, p53 signaling, MAP kinases, circadian rhythm, and cell cycle. For L-NSC: sensory perception of smell, mitochondrial organization, protein modification by small protein conjugation, cognition, neurologic system processes, neddylation, oxidative phosphorylation, G protein coupled receptor protein signaling, androgen and estrogen metabolism, ribosomes, translational elongation, and particularly noteworthy, pathways of Parkinson’s, Alzheimer’s and Huntington’s diseases. For MM6: modulation of P53 signaling, JAK-STAT signaling, programed cell death, arachidonic acid metabolism, response to hypoxia. Further, many of the perturbed miRNAs in the transcriptome have been associated with different cancer types, neurodegenerative diseases and the NFkB activation pathway. For instance, both infected S-NSC and S-NDC cells overexpressed mir-139, a microRNA that is overexpressed in the hippocampus of a mouse model for Alzheimer’s Disease (AD) and associated with impaired hippocampus-dependent learning and memory^57^. In addition, S-NSC cells infected with *T. gondii* PRU tachyzoites had more than a two-fold reduction in the expression of mir-29a and mir-107, found to be down-regulated in patients with AD^58–61^. mir-132, that is under-regulated in post-mortem Huntington’s disease patients and in a mouse model for this disease^62^, showed a ~four-fold down-regulation in GT1 and PRU-infected S-NSC cells. Expression of other miRNA molecules, that have been associated with the NFκB network and cancer, was also perturbed in S-NSC infected cells, such as mir-218, mir-143, mir-155, mir-199a, mir-21, miR-16 and mir-181b-1^63–71^.

In the quantitative proteomics with L-NSC (Fig. 5a), ATXN2L, FXR1, and NPC2 were modulated. ATXN2L is a paralog of Ataxin 2, a protein that causes spinocerebellar ataxia type 2^72^. It has been shown that ATXN2L is functionally similar to Ataxin 2 with respect to RNA metabolism and also plays a role in the regulation of stress granules and processing bodies in mammalian cells^73^. Overexpression of FXR1, a member of the Fragile X-related family of RNA-binding proteins, has been associated with suppression of cellular senescence and cancer^74^. NPC2 regulates the transport of cholesterol through the late endosomal/lysosomal system and mutations in this gene have been associated with Niemann-Pick disease type C2, a disease with a broad range of visceral, neurological and psychiatric clinical presentations^75^.

In the quantitative proteomics of S-NSC (Fig. 5b) many more genes transcripts were modulated. Some of particular interest are shown in Fig. 5b as follows: WDFY1 and PPP4C, two proteins known to modulate NFκB activity, a key factor to control parasite infections, were downregulated in infected S-NSC (Fig. 5b). WDFY1 induces TLR3- and TLR4-mediated activation of NFκB and the production of type I interferons and inflammatory cytokines^76^, while PPP4C is the catalytic subunit of protein phosphatase 4, a protein implicated in the activation of NFκB-mediated transcription^77^. This strongly suggests that *T. gondii* inhibits the human host NFkB pathway at multiple levels in addition to promoting p65 degradation through the virulence factor ROP 18^78^. Another two proteins, UBE3A and USP8, involved in protein ubiquitination, were also downregulated in infected S-NSC (Supplement B: Table S15). Interestingly, deubiquitin USP8 regulates the turnover of the epidermal growth factor receptor (EGFR) and its hyperactivation has been associated to constitutive EGFR-signaling leading to corticotroph tumorigenesis^79^. USP8 also regulates parkin-mediated mitophagy, a process believed to be central to the pathogenesis of Parkinson’s disease^80, 81^. Loss of function of the HECT-type E3 ubiquitin ligase UBE3A leads to Angelman syndrome, characterized by microcephaly, severe developmental delay, ataxia, seizures, and happy disposition^82^. Decrease expression of UBE3A has also been observed in Rett syndrome patients^83^. Within the proteins that were upregulated during S-NSC infection, was eEF1A2, a translation elongation factor that has been proposed to play a significant role in tumorigenesis and as an anti-apoptotic factor^84–86^ (Table S15). Pathways of cell death, TOR, protein transport/localization, RNA splicing, alternative splicing, oxidation reduction, vesicle mediated transport, iron homeostasis, glucose metabolism are noteworthy in this proteomics analysis.

We found it remarkable that we identified serum biomarkers in the ill children compared with those who were well (Fig. 6a–c). In considering these biomarkers in serum, three of those miRNAs, mir-17, mir-18b and mir-19a, are all encoded by the miR-17-92 family of miRNA clusters that modulate a number of protein-coding genes implicated in apoptosis, cell proliferation and angiogenesis^87^ (Fig. 6d). One of these miRs^88^, mir-17, is over-expressed in Human Foreskin Fibroblast cultures infected with the RH strain of *T. gondii*^89^. Also noteworthy, mir-124 associated with neurodegeneration^90^, was increased in sera of the three ill children (Fig. 6b,c). In addition, proteomics identified that ill children compared with their paired healthy controls had increases or decreases in certain serum proteins. Elevated proteins included clusterin (CLU)^91–93^, serum amyloid P-component (APCS)^94, 95^, and oxytocin(OXT)^96–99^ (Fig. 6d,e)^91, 92, 95, 100–106^. PGLYRP2 (Peptidoglycan recognition protein 2, with N-acetylmuramoyl-L-alanine amidase activity), that degrades an innate immunity recognition factor for peptidoglycans, was decreased in three of the ill children in the pairs (Fig. 6d). Apolipoprotein A1 also was decreased in the ill children (Fig. 6). These proteins are known to be associated with neurodegeneration. Specifically, clusterin is a chaperone which is increased in neurodegenerative diseases. It aids protein folding of secreted proteins, with three isoforms that are differently involved in pro- or anti-apoptotic processes. Thus this protein is involved in many diseases where there is oxidative stress including neurodegenerative diseases and aging. It is associated with Lewy bodies in Parkinson’s disease, with the pathology in Alzheimer’s disease and multiple system atrophy, Cerebrospinal fluid levels of clusterin may reflect pathology in neurodegenerative disease. Amyloid P is in amyloid fibrils and protects them from degradation, thus contributing to neurodegeneration in Alzheimer’s disease^94^. It is also an acute phase reactant. Oxytocin was present in the sera of the ill boys. Oxytocin diminishes inflammation, decreases anxiety, increases trust and empathy and mutations have been associated with autism spectrum disorder^96–99^. Hypothalamic cells produce oxytocin which is then secreted into the bloodstream by the posterior pituitary gland^96–99^. Secretion occurs when there is electrical activity, excitation, of hypothalamic neurons^96–99^. These findings suggest active brain destruction by the parasite or the response to it. These circulating miRNAs and proteins might prove clinically useful biomarkers to identify active toxoplasmic brain (or possibly retinal) disease if confirmed with more children’s sera correlated with their clinical findings.

Key upstream regulators were identified as shown in Figs. 7 and 8. Descriptions of target and upstream regulatory genes analyzed for L-NSC, cohort genetics and cohort biomarkers considered together are illustrated in Fig. 7. Key regulators we have found herein have been demonstrated to have significance in earlier work with other cell types: Those known empirically include HIF1α/VEGF^107, 108^ and others such as EGFR^109^ (Fig. 7; Details in Supplement B Table S7). IPA annotations of the total brain infectome and upstream regulatory bundle includes core (Supplement B: Table S7) and comparison (Supplement B: Table S7) analyses of target genes, upstream regulators and both. The convergence of the genes, biomarkers, trancriptomic data, proteomic data on the upstream regulators seen in this orbital diagram makes a model of the infectome which can be further empirically tested. *T. gondii* molecules which modify them then can be identified. Upstream regulatory genes such as *JUN, MYC, EGFR*, and *VEGF*^110^ provide examples of genes already known to be modulated by specific parasite proteins in other cell types^109, 111–113^. The biologic relevance of this finding for humans is evident from the observation that VEGF is very important in choroidal neovascular membranes that occur in clinical toxoplasmic chorioretinitis. These resolve when treated with antibody to VEGF administered in conjunction with anti-*T. gondii* medicines^108^.

Upstream regulatory genes contributing to the signature pathways with important biologic impact were compared when both L-NSC and S-NSC transcriptomic and proteomic data were analyzed together (Fig. 8). Noteworthy upsteam regulators were identified. For example, fibroblast growth factor^114^ and its receptors (e.g. FGRR1^115^), TGF-β, as well as the, ERK genes, PI3K, FoxOs^116^, and GM-CSF^47, 117^ are all involved in developmental and adult neurogenesis. Both TGF-b^118^ and PI3K/Akt^119^ are involved in ROS and inflammation-related actions during normal and pathological neurogenesis. In addition, TGF-β has been implicated in normal neural stem/progenitor cell growth and differentiation, as well as in cancer stem cell-mediated gliomagenesis^120^. Likewise, ERK signaling^121^ is involved in normal neural stem/progenitor cell fate choice during development. Another stem cell pathology involving PI3K/PTEN^122^ has shown that altering this pathway can result in interneuronal dysplasia and leukodystrophy as a result of altering neuronal and oligodendrocyte differentiation. All of these abnormal neurogenic phenotypes were present in the infected adult human neural progenitor cell population studied.

Cluster protein interactions of L-NSC total infectome provided profound insight into pathogenic mechanisms (Fig. 9). The functional clusters included cellular movement and migration important in mechanisms of immune and endocrine signaling, leucocyte migration, parasitism of lipid metabolism, ubiquitin-mediated protein degradation in cell cycle control, and hijacking protein synthesis in the cell cycle, all clustering around NEDD8^123–125^. As with ubiquitin and SUMO, NEDD8^123–125^ is conjugated to cellular proteins after the C-terminal tail is processed. *T. gondii* tachyzoites thereby alter host cell protein stability and degradation, potentially contributing to ER “stress” and the misfolded protein response associated with neurodegeneration. Other clusters include modulation of brain ATP production by mitochondrial oxidative phosphorylation. Another cluster shows effect on olfactory receptors (Fig. 9) suggesting a mechanism whereby parasites alter host sense of smell as seen in attraction of rodents and chimpanzees to cat urine^56, 126^. Parasitic modulation of these essential functions of the brains could lead to a wide range of diseases, including those discussed below (Fig. 10).

One NSC phenotype suggested by our genetics and omics analyses led us to study phenotypic effect of *T. gondii* isolates on NFκB in S-NSC with IFA (Fig. 11). Effect on NFκB noted in murine cell lines, macrophages and human fibroblasts and primary monocytes by others^34, 36^ were found mediated by Type II parasite dense granule protein (GRA) 15, whereas we found *T. gondii* infection of primary human neuronal stem cells by all three strains alters localization of NFκB (Fig. 11a). In other cell lines, STAT3 localization was modulated only by parasite Type I ROP16^127–129^. This is similar, but not identical, to the nuclear translocation of STAT3 in our human neuronal stem cells, which was increased most, but not exclusively, by the Type I strain. There was some similar effect for Type II and III strains (Fig. 11a). Effects on such cytokine signaling pathways have potential to contribute to maternal cytokine effects^130^ on fetal brain.

Another phenotype we selected for study included presence and localization of neurotransmitters (e.g., dopamine) and an enzyme in the brain involved in synthesis of this neurotransmitter, tyrosine hydroxylase. As shown in Fig. 11, we found *T. gondii* affects both dopamine and tyrosine hydroxylase and *T. gondii* tachyzoites contain dopamine. Disruption of neurotransmission is associated with epilepsy^5, 131^. Alteration of neurotransmitters is consistent with association studies of seropositivity and human epilepsy^131–134^, and three separate studies of mice^4, 5, 131^. These results are consistent with theories of *T. gondii*’s effects on reward pathways and depletion of serotonin by precursor tryptophan starvation^9, 135, 136^. These findings concerning neurotransmitters may support alterations of infectomes caused by the parasite affecting behavior, but these are predictions based on putative mechanisms observed in cells in tissue culture and remain to be confirmed empirically with experiments *in vivo*.

In other diseases, such as certain genetic diseases with repetitive DNA sequences, alternative splicing or mis-coding of transcripts can lead to truncated or misfolded proteins that are central causes of neurodegenerative diseases^137^. Perturbing genes associated with the misfolded protein response, and protein degradation seen in the IPA analyses, as well as inflammation are mechanisms whereby *T. gondii* may contribute to neurodegeneration, alterations of cell cycle/replication and death, and epilepsy^4, 133, 134, 138^ (Figs 10 and 11).

When we place our findings presented herein in the broad context of diseases and mechanisms of specific diseases, our work indicates *T. gondii* can cause a dominant alteration of the cell cycle and opposing regulation of cell growth and death. For example, as discussed above, in infected L-NSC, transcriptomes also showed that parasites modulate pathways of cell death, apoptosis and neddylation. Modulation in p53 signaling, ribosomes, amino acid metabolism, axon guidance, JAK-STAT, TGF-β, and cell cycle are especially noteworthy in the KEGG pathways for S-NSC (Fig. 2c). Transcriptomic analysis of L-NSC reveals pathways associated with Alzheimer’s, Parkinson’s and Huntington diseases and disruption of oxidative phosphorylation. Also alternative splicing pathways which might cause protein misfolding and neurologic diseases are affected (Fig. 5). Transcriptomics, proteomics, and cluster deconvolution identified alterations in neddylation and ubiquitination key in clearing misfolded proteins, neuronal cell viability, and synaptic plasticity (Figs 3–5, 9). We are investigating whether *T. gondii* causes alternative splicing which can be a central cause of protein misfolding, neurodegeneration, and other complex diseases^137^.

Although there are literature reports of associations between *T. gondii* seropositivity and schizophrenia in individual studies and in a meta-analyses of 38 studies^139^, there is no proven causality. An increase in dopamine metabolism is one possible pathologic mechanism^140, 141^. We detected dopamine and tyrosine hydroxylase immunostaining in the cytoplasm of *T. gondii* tachyzoites (Type I, II, and III) in S-NSC (Fig. 11). Alteration in dopamine neurotransmission has wide implications in behaviors and diseases, including epilepsy, neurodegeneration and movement disorders^142^. However, we do not identify any congenitally infected persons or their mothers with schizophrenia in our NCCCTS cohort (unpublished observations). Genetics and epigenetics of neuronal stem cells may be derived from donors who are not predisposed to schizophrenia. Primary cultures of NSC were temporal lobe tissues transected from patients who have epilepsy.

Cancer is the largest disease correlate to our *T. gondii* brain infectome. One explanation may be relative robustness of cancer research in comparison to other diseases in literature-based analyses. Some population studies show correlation with brain cancer. There are anecdotal descriptions of lymphomas developing in eyes of those with recurrent *Toxoplasmic* retinal disease^143, 144^. Targeted genetics studies of these *T. gondii* induced cancer genes and pathways might reveal higher penetrance. A strong argument for a *T. gondii*-cancer link is long known protection against tumor cells in murine models^145^. Recently, injection of attenuated, non-replicating parasites increased long-term survival of mice with melanoma^146^, pancreatic^147^, and ovarian^148^ cancers by stimulating high-level expression of co-stimulatory molecules CD80, CD86, IL-12, and tumor antigen specific CD8 + T cell populations and increasing cytolytic capacity of activated macrophages^149^. *T. gondii* may effect control of tumor growth and clearance through a network of 1,178 genes we have identified. Furthermore, our data may illuminate likely ways *T. gondii* may affect cancer stem cells, including stemness pathways of Wnt, TGF-β, STAT, among others^150^ and potential associations with Alzheimer’s disease. Some population serologic studies show conflicting correlations for^151, 152^ and against^153, 154^ *T. gondii* as a risk factor of Alzheimer’s disease or memory impairment. The parasite, however, inhibits neuronal degeneration as well as learning and memory impairments by immunosuppression in a murine model of Alzheimer’s disease^155^. *T. gondii* causes epilepsy^131^, possibly by altering GABAergic signaling^6^. Our analysis provides the systems map to study these correlations and applications of *T. gondii* as an immunotherapeutic tool.

Olfactory dysfunction is reported in Alzheimer’s disease and schizophrenia^156^. *T. gondii* increases cat predation of an infected rat by altering neural activity in limbic brain areas to block innate aversion of rats for cat urine. Infected chimpanzees lose their innate aversion towards urine of their natural predator, leopards^126^. There is a report that *T. gondii* might alter olfactory preferences in humans^157^. Our cluster deconvolution discovered 12 olfactory receptors in humans modulated by the brain parasite possibly causing olfactory preferences and dysfunction.

Only a subset of people with *T. gondii* infection, at the outset, have some of the diseases, like epilepsy or malignancy, that share the alterations in the signature pathways we identified. Our data indicate these signature, shared, molecular/cellular pathways, including inflammation, protein misfolding and mis-splicing, are altered by *T. gondii*. We found that effects differ in some cases for parasites with differing genetics and cells of different types or from different people. Our results provide insight into mechanisms whereby this parasite could cause these associated diseases under some circumstances. We show that tachyzoites, the rapidly growing form, can cause these^158^ alterations. *In vivo* this parasite interconverts from a dormant bradyzoite phase to an actively replicating tachyzoite phase and back again. This interconversion may be relevant to associations we recognized. Biomarkers we found were in ill children with new seizures and the two where we looked for this had reactivation of infection with activity documented at the time. In a separate parallel analysis of dormant parasites that form definitive cysts *in vitro*, similar pathways also were altered^159^ KEGG and GO analyses^159^ demonstrated a bradyzoite phenotype organism, called EGS, affects pathways involved in Alzheimer’s, Parkinson’s and Huntington’s diseases, splicing, and oxidative damage^159^. Thus, both tachyzoites and bradyzoites perturb critical host signaling pathways in common with those perturbed in epilepsy, neurodegenerative diseases, motor diseases including movement disorders, and brain cancer.

In neurodegenerative diseases and cancer, we have also shown glioma-initiating cancer stem-like cells possess an altered stem cell/developmentally regulated gene and protein mutanome^160–163^. An Adult Human Neural Progenitor Cell (AHNP)^49^, has been suggested to be susceptible to chronic microenvironmental inflammation in Parkinson’s Disease where it exhibits aberrant growth and differentiation^164^; in Alzheimer’s^165^, other neurodegenerative diseases and brain cancer. There are common growth-related programs and mutanomes in astrocytes and neurogenic astrocytes^166^ that are at-risk for transformation during chronic inflammation. Altered levels of cytokines and other immune- and inflammation-associated pathways, following *T. gondii* infection, have potential to support neurodegeneration and neoplastic transformation. It is notable that Zika virus infections also have a profound destructive effect on neuronal progenitor cells^167^ that share a normal or cancerous neuropoietic (i.e. persistent neurogenic) role during brain development and in the adult brain, including in the hippocampus^168, 169^. We have shown that pathology in this cell population is also involved in epilepsy, memory disorders, and autism in an interactome based on the literature. Carter *et al*. described similar overlap in neurologic susceptibility genes and those *T. gondii* modulates, based on a literature analysis. This created an interactome of *T.gondii* infection and genes implicated in a variety of neurologic and other diseases^9^. These diseases included multiple sclerosis, neurodegenerative diseases, epilepsy, and malignancies^9^. Overlap in Carter *et al*.’s analysis with those genes and pathways that *T. gondii* perturbs is striking^9^, although it is possible that they all involve the same pathways without *T. gondii* causing the diseases. A large data analysis of insured U.S. patients revealed the same associations with these diseases without clear directionality^10^.

All of these findings together support the notion that the dormant parasite, which sometimes interconverts to active tachyzoites when cysts rupture, and are present in chronic *T. gondii* infection in the brain of 2 billion persons, also has potential to contribute to these disease pathways. We identify some of the same mechanisms with tachyzoites. There is growing evidence for central nervous system transcellular spread of toxins, viruses, lectins and macromolecules including pathological proteins and nucleic acids. This serves to transmit such infectious elements^170, 171^ to naïve cells in neurodegenerative diseases (e.g., Parkinson’s and Alzheimer’s diseases) and cancer. In these diseases particular neural sites and associated circuitries are hijacked and also affect the same neural stem/progenitor cell studied here. We found in primary, *T. gondii* infected human neuronal stem cells these circuitries are altered in pathways of neurodegeneration, motor disease, movement disorders, epilepsy, malignancy and in odorant receptors. Neddylation, ubiquitination, alternative splicing, cell replication, and autophagy are altered, among others. These alterations provide some predicted mechanisms for the various clinical disease associations that also have been noted by others^51, 138, 172–181^.

Human chronic diseases are a complex interplay of genetic and environmental factors^9, 136, 182–184^ (Figs 1 and 10) requiring modified approaches to reconstruct their multifactorial etiology and cascades of developmental-plasticity mechanisms in precipitating disease. Human brain parasitism by *T. gondii* provides a model and template to examine development of brain diseases. This work provides a systems roadmap to design medicines and vaccines to repair and prevent neuropathologic effects of *T. gondii* infection of the human brain. Further, our original template provides a novel method to integrate multiple levels of intrinsic and extrinsic factors highlighting a way to unravel complexity in brain parasitism, toxoplasmosis specifically, and other complex diseases.

## Materials and Methods

### Genetics

#### Patient cohort and genotyping

Our approach to understand *T. gondii* brain infection (Fig. 1a) began with studies of families in which a child had congenital toxoplasmosis. We used clinical correlates and genetics approaches with data from our cohort, the National Collaborative Chicago Based Congenital Toxoplasmosis Study (NCCCTS). This program has diagnosed, treated and followed 246 congenitally infected persons and their families continuously beginning in 1981, and thereafter in an ongoing manner through the present. In this work, evaluations of affected persons took place from as early as gestation, when possible, through older adulthood. These evaluations were/are at regular pre-specified intervals with standardized protocols, in addition to other clinically indicated times. These evaluations occur in a single center in Chicago, performed by a single, consistent group of physician evaluators^11–39^. In our earlier, published work^11–39^, this cohort and method of analysis provided a powerful tool to identify genes and pathways causing susceptibility to toxoplasmosis (Table 1, Fig. 1). In this earlier work, human susceptibility alleles of candidate genes for those in the NCCCTS were identified using Transmission Disequilibrium Testing (TDT).

As described in our earlier work, we used samples from patient-parent trios (a congenitally infected individual and his/her biological parents) from the National Collaborative Chicago-Based Congenital Toxoplasmosis Study (NCCCTS)^12–14, 25, 31^. We extracted DNA from peripheral blood mononuclear cells (PBMC) obtained from 149 patient-parent groups that were genotyped at 12 single nucleotide polymorphism tags (tag-SNPs) throughout the specific gene. Tag-SNPs were then selected from the International HapMap Project, release 21 (http://www.hapmap.org)^12–14, 25, 31^. This was done using a 10-kb flanking sequence on either side of the gene^12–14, 25, 31^. A minor allele frequency (MAF) cutoff of 5% in Utah residents with Northern and Western European ancestry (CEU) and an r^2^ threshold of 0.8 were used^12–14, 25, 31^. Tag-SNPs were selected using the Tagger tool in the Haploview program^12–14, 25, 31^. UNPHASED-2.404 https://sites.google.com/site/fdudbridge/software/unphased-2-404 was used for the allelic association analysis of 124 infected children in the NCCCTS cohort who had a confirmed presentation of clinical disease involving the eyes and/or brain^12–14, 25, 31^. Hardy Weinberg Disequilibrium: The selected SNPs had an r^2^ threshold of 0.8, a minor allele frequency of >0.2, and were in Hardy-Weinberg equilibrium in the unrelated parents^12–14, 25, 31^. For the genetic study, allelic association analysis was performed using conventional TDT to determine the linkage disequilibrium (LD). LD is the nonrandom association of alleles at two or more loci^12–14, 25, 31^. It is essentially an approximation of the existence of historical recombination between 2 loci^12–14, 25, 31^.

#### NCCCTS informed consent and permissions

All studies involving human participants at the University of Chicago were approved by the University of Chicago IRB. Informed consent was obtained from all participants and/or their legal guardians. Informed consent to publish the image in Fig. 1b was obtained.

#### Statistical analysis for genetics

In the genetics study, allelic association was analyzed using a conventional transmission disequilibrium test (TDT), and *P* values were calculated using Haploview (http://www.broadinstitute.org/haploview), where *P* values less than or equal to 0.05 were considered significant for association with disease. P values are nominal and not Bonferroni corrected.

#### Analysis of Allen Brain Atlas Data for expresson of human toxoplasmosis susceptibility genes

The Allen Brain Atlas (http://human.brain-map.org) was used to search for each gene of interest. The expression of each gene was then visualized using the Allen Human Brain Atlas Brain Explorer 2 software (http://human.brain-map.org/static/brainexplorer). The expression of each gene in each region of the brain is shown using a heat map of the z-score for a specific probe for that gene. The z-score represents the normalized gene expression of that probe across the entire brain. We used the following colors to illustrate gene expression: Red represents increased expression and blue represents decreased expression.

### Cells, Culturing and Microscopy

#### L-NSC Cells

All human brain tissues used in these studies were obtained from the CPMC Neurosurgery Department, under an IRB approved protocol (Protocol #25.125-1). All patients provided written consent stating that they allowed for their samples to be used for basic research. The California Pacific Medical Center Institutional Review Board Panel#1 approved the tissue collection protocol, including the patient consent forms (Current IRB Assurance NO: FWA00000921). Samples were de-identified before being processed, to protect patient privacy. The L-NSC cell line was derived from the hippocampus tissue removed from a patient with intractable epilepsy. Cells were characterized by immunofluorescence and found positive for the neural stem and progenitor cell markers Nestin, glial fibrillary acidic protein or GFAP, Beta-III tubulin or Tuj1 as a neuronal marker, and Olig 2 as a neuronal and glial progenitor cell marker. All experiments were performed on passages 2–5 from the NPC culture. This was discussed previously in http://cancerres.aacrjournals.org/content/early/2011/09/06/0008-5nfk2.CAN-11-07. All methods were performed in accordance with the relevant guidelines and regulations.

#### S-NSC Cells

IRB-approved and consented, from the University of Florida, S-NSC cells are adult human neural stem/progenitor cells originally referred to as adult human neural progenitor cells or “AHNP” cells, and they were cultured here according to the published protocol^49^. The culture conditions and nomenclature are shown in 2.

#### Human monocytic cell line

*In vitro* culture-adapted human monocytes, i.e., MonoMac6 cells^185^, were used. MonoMac6 cells were seeded at 4 × 105/well in a 24-well plate in RPMI medium supplemented with 10% fetal calf serum, 2.05 mM L-glutamine, 1 × nonessential amino acids (Sigma), OPI medium supplement (Hybri Max; Sigma), and 1% penicillin-streptomycin (Thermo Fisher).

### Toxoplasma gondii

*Toxoplasma gondii* type I (GT1, RH), type II (Me49; Pru), and type III (VEG) maintained in HFF were used to infect the cells at a m.o.i. of 4, and incubated for 18 hrs. Extracellular *Toxoplasma gondii* were removed by washing with cold PBS before cells were harvested for total RNA extraction or IFA. Specific parasites used in each experiment are specified in the results and figures.

#### Immunostaining and IFA

For immunostaining study, AHNP (S-NSC) cells were grown on glass cover slip before infected with *Toxoplasma gondii*. 24 hours after infection, cells were fixed with paraformaldehyde and standard immunefluroescent staining protocol was adopted for studying the localization of targets of interest, including NFκB, STAT3, tyrosine hydroxylase, and dopamine. Antibodies utilized include: anti-NFκB p50 (Santa Cruz Biotech, SC-7178); P-STAT3 (Y705), cell signaling (#91315); anti-tyrosin hydroxylase (Sigma, T1299); and dopamine antibody (Novus Biologicals, NB120-1001).

### RNA isolation, quantitation: RNA purification, RNA microarray and bioinformatics, RNA sequencing and bioinformatics, miR sequencing

#### Isolation of RNA

Type I-II-III single celled organisms were grown in S-NSC, S-NDC or MM6. RNA was isolated and processed for sequencing as described. Below. In all cases purified RNA had RIN scores >8. All experiments were done using biological replicates.

#### Affymetrix chip

Gene expression profiling was performed using the Affymetrix GeneChip® Human Gene 1.0 ST Array (Affymetrix Inc., Santa Clara, CA). Total RNA was isolated using miRNeasy Mini Kit (Qiagen), and converted to cDNA following the Ambion WT Expression protocol. Briefly, total RNA (250 ng) was used for 1st and 2nd strand cDNA synthesis. cRNA was obtained by an *in vitro* transcription reaction, and was then used as the template for generating 1st strand cDNA. The cDNA was fragmented and end-labeled with biotin using the GeneChip® WT Terminal Labeling kit. The biotin labeled cDNA was hybridized to the array for 16 hours at 45 °C using the GeneChip® Hybridization Oven 640. Washing and staining with streptavidin-phycoerythrin was performed using the GeneChip® Hybridization, Wash and Stain Kit, and the GeneChip® Fluidics Station 450. Images were acquired using the GeneChip® Scanner 3000 7 G and the GeneChip® Command Console® Software (AGCC).

#### Differential gene and miRNA expression analysis

Total RNA samples extracted from uninfected human cell cultures (controls) or from the same human cells infected with type I, II or III *T. gondii* strains for 18 hours, were treated with miRNeasy Mini Kit columns (Qiagen) following manufacturer instructions in order to separate both mRNA and miRNA fractions for each condition. Thereafter, Illumina barcoded sequencing libraries were constructed with TruSeq RNA Sample Preparation Kits v2 (Illumina) or NEBNext Multiplex Small RNA Library Preparation Kit (NEB) for mRNA or miRNA sequencing respectively. All sequencing libraries were sequenced using Illumina HiSeq. 2000 technology (100 bp single-end reads) and multiplexed in groups of 6 libraries per lane for mRNA (total sequencing data per mRNA sample ~3 Gb) and 9 libraries per lane for miRNA (total sequencing data per miRNA sample ~2 Gb). To quantify protein coding gene expression, CLC Genomic Workbench software (CLC Bio-Qiagen, Aarhus, Denmark) was used to map sequencing reads to the human reference assembly (release GRCh38) and the *T. gondii* ME49 reference genome (ToxoDB release 13.0). Afterwards, to identify genes that were differentially expressed in infected cultures compared to their respective uninfected control samples, read counts per gene were analyzed with the R package edgeR using a generalized linear model likelihood ratio test.

On the other hand, to assess miRNA differential gene expression, miRNA sequencing reads were first depleted of adaptor and primer sequences and then mapped with CLC Genomic Workbench software to the human reference genome assembly (GRCh38) using the miRNA annotations from miRBase v21 (www.mirbase.org). Identification of human miRNA genes that were differentially expressed in infected host cells compared to their corresponding uninfected controls was carried out with edgeR using a generalized linear model likelihood ratio test.

For both mRNA and miRNA analyses, p-values were adjusted for multiple hypotheses testing using the False Discovery Rate method. MDS plots and heat maps were generated with the plotMDS tool from edgeR and the R tool heatmap. Differentially expressed genes in MM6 and NSC cell lines infected with Type I-II-III parasites were identified under the criteria of 1% FDR and absolute log2-fold-change >1.5 (i.e. fold-change >2.8 and <0.35 for up- and down-regulated genes, respectively).

#### Proteomics

The protocol was developed in 6 steps: (1) protein extraction with urea and SDS (2) protein digestion with Lys-C/ trypsin, (3) peptide labeling with isobaric iTRAQ reagents, (4) peptide fractionation by isoelectric point, (5) peptide identification and relative quantitation by tandem mass spectrometry with “higher energy collision-induced dissociation” (HCD), and (6) bioinformatic analysis of mass spectroscopic data.

#### L-NSC Quantitative Proteomic using Multiplexed Isobaric Tandem Mass Tags

The protocol for Protein quantification, Protein extraction, iTRAQ labeling and mass spectrometry, Bioinformatics, and System Bioinformatics was developed in six steps:

Step 1. Infected (RH, ME49, VEG strains of *Toxoplasma gondii*) and uninfected human NPCs were denatured in 8 M Urea/ 0.4 M ammonium bicarbonate buffer, pH 8.0, and the urea soluble fraction was collected by centrifugation. Membrane proteins were extracted from the urea-insoluble pellet by adding 2% SDS to a final concentration of 0.125% (vol/vol) SDS. This sample fractions were then sonicated six times on ice with two to three second bursts followed by a thirty second cooling period, using a Sonic Dismembrator 60 (Fisher Scientific). After centrifugation this second supernatant was pooled with the first protein extract. The protein concentration in the pooled urea/ SDS solubilized protein extracts was determined using a BCA assay (control, RH, ME49, and VEG samples were 0.34, 0.49, 0.55 and 0.48 μg/uL, respectively).

Step 2. From this point, sample processing was performed in triplicate. For each sample, an aliquot containing 100 μg of total protein was added to Eppendorf tubes. A ten microliter aliquot of a solution of MassPREP Protein Standard Mix (Waters, P/N 186004900) was added to each to serve as a process control. Next, proteins were precipitated using a cold acetone/TCA procedure. Samples were resuspended in 8 M urea/ 0.4 M ammonium bicarbonate buffer (pH 8). After reduction and alkylation with dithiothreitol and iodoacetamide, the samples were diluted to 2 M urea/ 100 mM -ammonium bicarbonate, pH 8.0, final concentration. Lys-C was added at 1:200 (w/w) Lys-C:protein ratio and allowed to digest for 4 hours at 37 °C. Then Trypsin (Promega Gold) was added at 1:200 (w/w) and the digestion was allowed to digest overnight for approximately 16–20 hours total.

Step 3. After the proteolytic digestion, samples were desalted on a peptide macro trap (Michrom BioResources) and dried by vacuum centrifugation. Steps given in Applied Biosystems’ iTRAQ reagent protocol guide (part# 4350831 Rev. C) titled *Applied Biosystems iTRAQ Reagents: Amine-Modifying Labeling Reagents for Multiplexed Relative and Absolute Protein Quantitation* were followed for isobaric peptide labeling. First the samples were dissolved in 40 μL of the dissolution buffer from the iTRAQ buffer kit. The tryptic peptides in each sample were then labeled by the four tags (114: uninfected control; 115: RH; 116: ME49; 117: VEG) of a 4-plex iTRAQ kit. Isopropanol was used to dissolve the iTRAQ reagents instead of ethanol. After labeling, the four samples for each replicate were combined into a single Eppendorf tube. The samples were again dried by vacuum centrifugation prior to removal of excess iTRAQ reagent by strong cation exchange chromatography, again as per the vendor’s instructions. Finally, the samples were desalted again using a peptide macro trap in preparation for OffGel fractionation.

Step 4. The pooled labeled peptides were fractionated in-solution with an Agilent 3100 OFFGEL fractionator following the manufacturer’s recommendations (Agilent Technologies)^186, 187^. A set-up was used to separate peptides according to their isoelectric point, consisted of a 13 cm IPG strip pH 3–10, and IPG buffer, pH3-10 (GE Healthcare), and a 12 well frame set (Agilent Technologies). The unit was operated according to the Agilent’s Operator Guide.

Step 5. OffGel Fractions were analyzed by nano-flow liquid chromatography tandem mass spectrometry (LC/MS/MS). A nanoelectrospray source coupled an Ultimate 3000 LC system to a LTQ Orbitrap Velos Pro mass spectrometer (Thermo Fisher Scientific, Bremen Germany). During the injection sequence, peptides were concentrated on a Dionex u-Precolumn Acclaim PepMap100 (C18, 5 um, 100 A, 300 um × 5 mm) and washed/desalted for twenty minutes. Peptides were separated on a Zorbax 300 SB C18 (3.5 um, 150 mm × 75 um) nano-column from Agilent using a multi-step gradient spanning 100 minutes. Xcalibur was used to operate the system in data-dependent acquisition mode. The ten most intense peptide ions detected by the orbital analyzer were sequentially isolated for fragmentation in the HCD cell.

Step 6. We analyzed three analytical replicates for each of the four samples. Mass spectral data from the analysis of 12 IEF fractions were converted to MGF file formats using MassMatrix^188^, concatenated and submitted as a single large MGF file for searches against NCBI human and *Toxoplasma gondii* databases using a Mascot 2.2 (Matrix Science) search engine^189^. The following search parameters were used: (i) enzyme, trypsin; (ii) one missed cleavage allowed; (iii) fixed modifications, iTRAQ 4-plex, carbamidomethylation of cysteines; (iv) variable modification, oxidation of methionine; (v) peptide tolerance, 10 ppm; and (vi) MS/MS tolerance, 30 ppm. Mascot result files (.dat) were imported into Scaffold Q3 + (Proteome Software). Protein reporting thresholds were 95% protein probability, 90% peptide probability, with at least two peptides per protein. Algorithms embedded in Scaffold Q3 + were used to extract and to report the quantitative data collected during the iTRAQ experiment.

### S-NSC- proteomics

Worksheet “*Sample Proc*” summarizes the sample prep process. From each flask, ~180–190 ug proteins were extracted and 50 ug were used for 8-plex iTRAQ analysis. Worksheet “*Pep Ratio*” is the raw table listing relative ratios for all peptide identified in all 8 samples. The ratio should be 0.125 (1.000/8) if one peptide/protein evenly distributes among 8 samples. Ratios of peptides from the same proteins are then calculated to represent protein ratios. Worksheet “*Ratio to Channel 0*” includes a total of **4,367** identified proteins with iTRAQ ratio. Note the protein ratios across 8 samples (4 conditions in duplicates) are raw data from mass spectrometry and converted to ratios against Channel 0, *i.e*. Control sample 1. The data can also be normalized using the factors in Column O in worksheet “*Sample Proc*”. Columns X-Z are average ratios in each infection condition *versus*. average of controls. Worksheet “*Prot with high score*” has **3,359** proteins identified by more than 1 peptide and with ProteinProphet probability >0.8 (=FDR < 1%). Among these 3,359 proteins with high confidence, 10 protein concentrations increased by >2-fold in either of the 3 infected cells *vs*. controls, while 28 proteins decreased by >2-fold.

### Human serum samples used in biomarker discovery

These data were obtained as follows: Three pairs of children called pairs A, B, C were studied. They are described with additional information in the legend of Fig. 4a, and in the methods, in each demographically-matched pair, one child had severe disease and the other had mild or no clinical illness. Each child had serum stored from evaluations at the same ages, 3.5 years old (pairs A and C). The first pair (A1/A2) were Caucasian children of the same ethnicity, originally primarily British, background, and similar demographics. The second pair (B1 and B2) were of Caucasian/Filipino ethnicity, dizygotic, discordant twins who live in the same household. The third pair (C1 and C2) were children of Hispanic ethnicity from the same geographic regions and with similar demographic characteristics. Each clinically well child was identified because he was similar to the ill child. This was possible because we have in depth knowledge of the clinical findings and demographics of the children followed in the NCCCTS. Thus, it was possible to identify demographically matched well children for two (pair A and C) and a well twin for the ill twin (pair B). Serum Collection for Children in the NCCCTS is as follows. These children have serum prepared from blood drawn at each visit and the sera herein were obtained at a visit when new seizures were noted for ill children. The global proteome and miRNAome changes in sera were analyzed with iTRAQ and miRNA qPCR panels.

### Bioinformatics and Additional Systems Biology Analyses

The following analyses were performed at the Institute of Systems Biology and J. Craig Venter Institute:

#### DEGs and DEmiR analysis of effect of cell type and parasite type

Differentially expressed genes and proteins (iTRAQ) and human neuronal cells, culture conditions, and markers. A summary of DEG and DEmiRs. For RNAs, lowly expressed RNAs were removed; threshold was defined in #3 & #4 f. Distribution of read counts mapped to miRNAs. P < 0.01 and absolute log2-fold-change >1 (fold change >2 or <0.5). Relationship of differentially expressed genes (DEGs) by cell line, relationship of DEGs by strain, relationship of differentially expressed miRNAs (DEmiRs) by cell line, and relationship of DEmiRs by strain and functional enrichment analysis included. For identified DEGs, GO Biological Processes were enriched with DAVID software v6.7. GO Biological Processes with p-value < 0.01 and number of genes associated with certain GO term > = 5 summarized. KEGG pathway enrichment analysis is shown. For identified DEGs, KEGG pathways were enriched with DAVID software v6.7. KEGG pathways with p-value < 0.05 summarized.

### Upstream regulators for L-NSC and S-NSC transcriptomics and proteomics combined (ISB)

The upstream regulator analyses was performed to identify molecules regulating genes and proteins identified from transcriptomic and proteomic data for L-NSC or S-NSC.

Genes and proteins differentially expressed in L-NSC or S-NSC were combined, respectively and used as an input for the analysis. Based on the p-value < 0.01, 913 and 83 molecules were identified as upstream regulators of genes or proteins differentially expressed in S-NSC and L-NSC, respectively. Using interactions between 22 common upstream regulators and their target genes/proteins, gene regulatory networks were constructed using Cytoscape.

The Reconstruction-Deconvolution analysis was completed at BrainMicro LLC using Ingenuity Pathway Analysis tool (IPA, Inc.)^190, 191^ and STRING v9.1 web-tool^53^. Allen Atlas and IPA used human *T. gondii* susceptibility genes. They show specific genes expressed in the brain. Additionally, it includes upstream regulators found in IPA analysis. p-value of overlap < 5.0 × 10^−3^. Biomarkers for 3 ill children compared to well controls were then added.

STRING database provided analyses that are concentrated more on protein-protein interactions (Fig. 9; Supplement 6). IPA provided the core analysis of network annotations, canonical pathways (Supplement B: Tables S6, 9, 10), and disease-functions (Fig. 6; Supplement B:Table S6). In addition, IPA core analysis could detect predicted upstream regulators of gene networks that were potentially not captured in the snapshots of experimental design and data collection (Figs 1–6; Supplement B: S2, 7). In some cases when a larger dataset was parsed into smaller networks, a secondary IPA core analysis was completed to identify the properties of the segregated datasets. Top-scored IPA analysis was also used to condense the large annotation prediction for comparison. In these cases, only the top 10–25 pathways/networks with lowest P-value, highest Z-scores are shown (e.g., Fig. 4, Supplement B: Table S3). Due to the large number of citations for function of genes and pathways, when citations were not used, the information was obtained from the Ingenuity Knowledge Base (http://www.ingenuity.com/science/knowledge-base). Cut-off p-value for each analysis were shown in each analysis.

We approached the Reconstruction-Deconvolution analysis without any preconceptions. Our deep understanding in parasitology and biological processes were used to understand the significant patterns emerged with highest statistical values. Reconstruction of the brain infection used the template in Fig. 1. We examined the 1^st^ ‘layer’ (susceptibility genetics) by IPA for 3 features: canonical pathways, upstream regulation, and disease-functions. Each ‘layer’ of datasets was added sequentially in the following order of NSC transcript, NSC protein and toxoplasmosis biomarkers. Each new composite layer was thorough analyzed for the 3 IPA features to see the additive effects. Datasets were also segregated and analyzed separately by Type I, II and III infections. The upstream regulators were further analyzed separately for canonical pathways and disease-functions. It was important to have a complete, unbiased view of how each layer of data contributes and connects to each other. Only selected, important observations were described in this report.

The total brain infectome included all 4 ‘layers’ and segregated according to parasite strains (Supplement B: Table S7). Our Deconvolution strategy entailed 3 approaches. “Orbital Deconvolution’ was the visual design to show connections between the 4 layers including the upstream regulators (Fig. 7) and portion of IPA analysis is shown (Supplement B: Table S7). “Cluster Deconvolution’ utilized STRING to analyze meaning brain infectome of L-NSC, meaning that S-NSC data was excluded, due to the number limit of STRING interactions (Fig. 9, Supplement B: Table S6). We detected the 7 clusters based on visual detection of network maps. Genes for each cluster was analyzed for the 3 IPA features to determine their properties. “Disease Deconvolution” was derived from the IPA disease-functions analysis of the total brain infectome and graphically drawn in IPA (Fig. 10, Supplement B: Tables S10A, B).

#### Analysis of Alternatively Spliced Genes

To identify potential candidate protein coding genes that were differentially spliced between infected and uninfected S-NSC cells, mRNA sequencing reads derived from infected and uninfected S-NSC were mapped to the human reference assembly (GRCh38) with T*ophat*. Thereafter, mapping data were processed with rMATS^51^ to identify candidate alternative-spliced transcripts. Predicted alternative-spliced isoforms that were over- or under-represented in infected S-NSC cells compared to their respective controls with an adjusted p-value < 0.1 (false discovery rate method) were kept for future analyses(Accession numbers in Supplement D).

## Electronic supplementary material

Supplement C Part 1

Supplement C Part 2

Supplement B Part 1

Supplement B Part 2

Supplement A

Supplement D
