# Publisher Correction: *Toxoplasma* Modulates Signature Pathways of Human Epilepsy, Neurodegeneration & Cancer

**DOI:** 10.1038/s41598-019-44545-0

**Published:** 2019-05-28

**Authors:** Huân M. Ngô, Ying Zhou, Hernan Lorenzi, Kai Wang, Taek-Kyun Kim, Yong Zhou, Kamal El Bissati, Ernest Mui, Laura Fraczek, Seesandra V. Rajagopala, Craig W. Roberts, Fiona L. Henriquez, Alexandre Montpetit, Jenefer M. Blackwell, Sarra E. Jamieson, Kelsey Wheeler, Ian J. Begeman, Carlos Naranjo-Galvis, Ney Alliey-Rodriguez, Roderick G. Davis, Liliana Soroceanu, Charles Cobbs, Dennis A. Steindler, Kenneth Boyer, A. Gwendolyn Noble, Charles N. Swisher, Peter T. Heydemann, Peter Rabiah, Shawn Withers, Patricia Soteropoulos, Leroy Hood, Rima McLeod

**Affiliations:** 1 The University of Chicago, Chicago, IL 60637 USA; 2 Northwestern University, Feinberg School of Medicine, Chicago, IL 60611 USA; 3 BrainMicro LLC, New Haven, CT 06511 USA; 4 J Craig Venter Institute, Rockville, MD 20850 USA; 5 Institute for Systems Biology, Seattle, WA 98109 USA; 6 University of Strathclyde, Glasgow, G1 1XQ United Kingdom; 7 Genome Quebec, Montréal, QC H3B 1S6, Canada; McGill University, Montréal, QC H3A 0G4 Canada; 8 Department of Pathology, University of Cambridge, Cambridge, CB2 1QP United Kingdom; 9 Telethon Kids Institute, The University of Western Australia, Perth, Australia; 10 University of Illinois-Chicago, Chicago, IL 60607 USA; 11 California Pacific Medical Center, San Francisco, CA 94114 USA; 12 JM USDA Human Nutrition Research Center on Aging, Tufts University, Boston, MA 02111 USA; 13 Rush University Medical Center, Chicago, IL 60612 USA; 14 Northshore University Health System, Evanston, IL 60201 USA; 15 Rutgers University, Newark, New Jersey 07101 USA; 16 FLH, IBEHR School of Science and Sport, University of the West of Scotland, Paisley, PA1 2BE UK

Correction to: *Scientific Reports* 10.1038/s41598-017-10675-6, published online 13 September 2017

In the original version of this Article, the author Kamal El Bissati was incorrectly indexed. This error has now been corrected.

