# Supplementary material for: Toxoplasma Modulates Signature Pathways of Human Epilepsy, Neurodegeneration & Cancer
Source: Sci Rep. 2017 Sep 13;7:11496. doi: 10.1038/s41598-017-10675-6 (PMC5597608; doi:10.1038/s41598-017-10675-6)
Supplement: Supplementary file 1 — Supplement C Part 1 [file 41598_2017_10675_MOESM1_ESM.pdf]

Supplement C

***Toxoplasma* Modulates Signature Pathways of Human Epilepsy, Neurodegeneration & Cancer**

Huân M. Ngô[1,2,3,+], Ying Zhou[1,+], Hernan Lorenzi[4,+], Kai Wang[5,+], Taek-Kyun Kim[5,+], Yong Zhou[5], Kamal El Bissati[1], Ernest Mui[1], Laura Fraczek[1], Seesandra V. Rajagopala[4], Craig W. Roberts[6], Fiona L. Henriquez[1], Alexandre Montpetit[7], Jenefer M. Blackwell[8,9], Sarra E. Jamieson[9], Kelsey Wheeler[1], Ian J. Begeman[1], Carlos Naranjo-Galvis[1], Ney Alliey-Rodriguez[1], Roderick G. Davis[10], Liliana Soroceanu[11], Charles Cobbs[11], Dennis A. Steindler[12], Kenneth Boyer[13], A. Gwendolyn Noble[2], Charles N. Swisher[2], Peter T. Heydemann[13], Peter Rabiah[14], Shawn Withers[1], Patricia Soteropoulos[15], Leroy Hood[5], and Rima McLeod[1*]

[1]The University of Chicago, Chicago, IL 60637
[2]Northwestern University, Feinberg School of Medicine, Chicago, IL 60611
[3]BrainMicro LLC, New Haven, CT 06511
[4]J Craig Venter Institute, Rockville, MD 20850
[5]Institute of Systems Biology, Seattle, WA 98109
[6]University of Strathclyde, Glasgow G1 1XQ, United Kingdom
[7]Genome Quebec, Montréal, QC H3B 1S6, Canada; McGill University, Montréal, QC H3A 0G4, Canada
[8]Cambridge Institute for Medical Research and Department of Pathology, University of Cambridge, Cambridge CB2 1QP, United Kingdom
[9]Telethon Kids Institute, The University of Western Australia, Perth, Australia
[10]University of Illinois-Chicago, Chicago, IL 60607
[11]California Pacific Medical Center, San Francisco, CA 94114
[12]JM USDA Human Nutrition Research Center on Aging, Tufts University, Boston, MA 02111
[13]Rush University Medical Center, Chicago, IL 60612
[14]Northshore University Health System, Evanston, IL 60201
[15]Rutgers University, Newark, New Jersey 07101

[+] Equal contributions

* To whom Correspondence should be addressed:
Rima McLeod, M.D.
rmcleod@uchicago.edu

Co-Corresponding Authors:
Huân M. Ngô (h-ngo@northwestern.edu)
Hernan Lorenzi, PhD (hlorenzi@jcvi.org)
Kai Wang, PhD (kai.wang@systemsbiology.org)
T.K. Kim, MS (tkim@systemsbiology.org)

Current Addresses:
FLH, IBEHR School of Science and Sport, University of the West of Scotland, Paisley, PA1 2BE, UK

Supplemental Figure Legend

Figure S1. MDS plot of RNA-seq profiles of uninfected MM6, S-NSC and S-NDC cells.
