# Supplementary figures and images for: Toxoplasma Modulates Signature Pathways of Human Epilepsy, Neurodegeneration & Cancer
Source: Sci Rep. 2017 Sep 13;7:11496. doi: 10.1038/s41598-017-10675-6 (PMC5597608; doi:10.1038/s41598-017-10675-6)

# MDS of uninfected MM6, S-NSC and S-NDC mRNA-seq

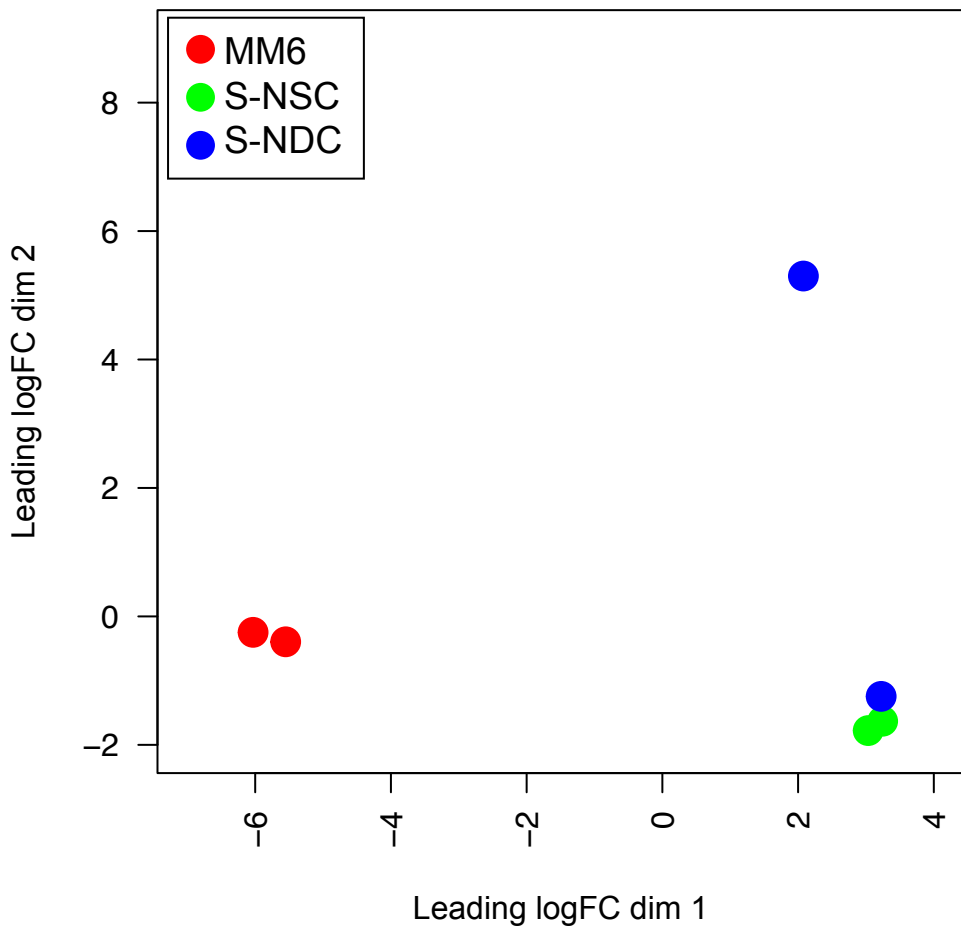

Supplement: Supplementary file 2 — Supplement C Part 2 [file 41598_2017_10675_MOESM2_ESM.pdf]
