# Supplementary material for: Toxoplasma Modulates Signature Pathways of Human Epilepsy, Neurodegeneration & Cancer
Source: Sci Rep. 2017 Sep 13;7:11496. doi: 10.1038/s41598-017-10675-6 (PMC5597608; doi:10.1038/s41598-017-10675-6)
Supplement: Supplementary file 3 — Supplement B Part 1 [file 41598_2017_10675_MOESM3_ESM.pdf]

Supplement B

***Toxoplasma* Modulates Signature Pathways of Human Epilepsy, Neurodegeneration & Cancer**

Huân M. Ngô[1,2,3,+], Ying Zhou[1,+], Hernan Lorenzi[4,+], Kai Wang[5,+], Taek-Kyun Kim[5,+], Yong Zhou[5], Kamal El Bissati[1], Ernest Mui[1], Laura Fraczek[1], Seesandra V. Rajagopala[4], Craig W. Roberts[6], Fiona L. Henriquez[1], Alexandre Montpetit[7], Jenefer M. Blackwell[8,9], Sarra E. Jamieson[9], Kelsey Wheeler[1], Ian J. Begeman[1], Carlos Naranjo-Galvis[1], Ney Alliey-Rodriguez[1], Roderick G. Davis[10], Liliana Soroceanu[11], Charles Cobbs[11], Dennis A. Steindler[12], Kenneth Boyer[13], A. Gwendolyn Noble[2], Charles N. Swisher[2], Peter T. Heydemann[13], Peter Rabiah[14], Shawn Withers[1], Patricia Soteropoulos[15], Leroy Hood[5], and Rima McLeod[1*]

[1]The University of Chicago, Chicago, IL 60637
[2]Northwestern University, Feinberg School of Medicine, Chicago, IL 60611
[3]BrainMicro LLC, New Haven, CT 06511
[4]J Craig Venter Institute, Rockville, MD 20850
[5]Institute of Systems Biology, Seattle, WA 98109
[6]University of Strathclyde, Glasgow G1 1XQ, United Kingdom
[7]Genome Quebec, Montréal, QC H3B 1S6, Canada; McGill University, Montréal, QC H3A 0G4, Canada
[8]Department of Pathology, University of Cambridge, Cambridge CB2 1QP, United Kingdom
[9]Telethon Kids Institute, The University of Western Australia, Perth, Australia
[10]University of Illinois-Chicago, Chicago, IL 60607
[11]California Pacific Medical Center, San Francisco, CA 94114
[12]JM USDA Human Nutrition Research Center on Aging, Tufts University, Boston, MA 02111
[13]Rush University Medical Center, Chicago, IL 60612
[14]Northshore University Health System, Evanston, IL 60201
[15]Rutgers University, Newark, New Jersey 07101

[+] Equal contributions

* To whom Correspondence should be addressed:
Rima McLeod, M.D.
rmcleod@uchicago.edu

Co-Corresponding Authors:
Huân M. Ngô (h-ngo@northwestern.edu)
Hernan Lorenzi, PhD (hlorenzi@jcvi.org)
Kai Wang, PhD (kai.wang@systemsbiology.org)
T.K. Kim, MS (tkim@systemsbiology.org)

Current Addresses:
FLH, IBEHR School of Science and Sport, University of the West of Scotland, Paisley, PA1 2BE, UK

Index to Supplementary Tables

Table S1. List of Identified Human Genes That Are Likely to Play a Role in Susceptibility to *T. gondii* Infection.

Table S2A. Analysis of Expression of Susceptibility Genes in Human Brain Using Allen Brain Atlas.

Table S2. Predicted Upstream Regulators of Congenital Toxoplasmosis Genetics and Biomarkers.

Table S3. Transcriptomics profiles of neuronal stem cells with significant modulation [≥ (+/-) 1.5 log2 fold change infected/control] due to *Toxoplasma* infection. RNA Quantification of L-NSC was determined by Affymetrix Gene Microarray, whereas S-NSC was assayed by RNA sequencing.

Table S4. Quantification of Proteins in Human L-NSC Infected with *Toxoplasma gondii* Type I, II or III for 18 Hours.

Table S5. Complete Assembly of Systemic Infectome in L-NSC and S-NSC by Gene Symbols.

Table S6. Protein-Protein Interactions as Determined by STRING Database in L-NSC and S-NSC.

Table S7. Upstream Regulation Analysis of Molecules Regulated by Infection in NSC cells and CT Cohort Genetic & Biomarkers. A, Upstream Regulatory Pathways from Figure 7; B, Gene Description of Upstream Regulator and Targets from Figure 7; C, Upstream Regulatory Pathways.

Table S8. Canonical Pathway Analysis.

Table S9. Canonical Pathways that includes NFKB1 as determined by IPA.

Table S10A. IPA Annotation of Diseases and Function.

Table S10B. IPA Annotation of Diseases and Function (Complete Annotation).

Table S11. microRNAs in Steindler neural stem and differentiated cells with significant modulation [FDR < 0.05] due to *Toxoplasma* infection. MiRNA quantification was assayed by RNA sequencing.

Table S12. microRNAs, grouped by human mature microRNA gene, with significant modulation [FDR < 0.05] due to *Toxoplasma* infection in MonoMac6 cells and Steindler neural stem and differentiated cells. MiRNA quantification was assayed by RNA sequencing. Reads are considered only if they map to one type of microRNA.

Table S13. mRNAs in MonoMac6 (MM6) cells with significant modulation [> (+/-) 1.5 log2 fold change] due to *Toxoplasma* infection. Quantification of mRNA was assayed by RNA sequencing.

Table S14: rMATS prediction of alternatively spliced genes in Steindler Neuronal Stem Cells infected with Type I, II and III *T. gondii* parasites using reads spanning splicing junctions and exon sequences.

Table S15: iTRAQ quantitative proteomics analysis of S-NSC infected with Type I, II and III *T. gondii* parasites.

Table S16: GO Biological Process enrichment analysis on MM6, S-NSC and S-NDC transcriptomics data.

Table S17: KEGG Pathways enrichment analysis on MM6, S-NSC and S-NDC transcriptomics data.
