# Supplementary material for: Toxoplasma Modulates Signature Pathways of Human Epilepsy, Neurodegeneration & Cancer
Source: Sci Rep. 2017 Sep 13;7:11496. doi: 10.1038/s41598-017-10675-6 (PMC5597608; doi:10.1038/s41598-017-10675-6)
Supplement: Supplementary file 5 — Supplement A [file 41598_2017_10675_MOESM5_ESM.pdf]

Supplement A

Toxoplasma Modulates Signature Pathways of Human Epilepsy, Neurodegeneration & Cancer

Huân M. Ngô[1,2,3,+], Ying Zhou[1,+], Hernan Lorenzi[4,+], Kai Wang[5,+], Taek-Kyun Kim[5,+], Yong Zhou[5], Kamal El Bissati[1], Ernest Mui[1], Laura Fraczek[1], Seesandra V. Rajagopala[4], Craig W. Roberts[6], Fiona L. Henriquez[1], Alexandre Montpetit[7], Jenefer M. Blackwell[8,9], Sarra E. Jamieson[9], Kelsey Wheeler[1], Ian J. Begeman[1], Carlos Naranjo-Galvis[1], Ney Alliey-Rodriguez[1], Roderick G. Davis[10], Liliana Soroceanu[11], Charles Cobbs[11], Dennis A. Steindler[12], Kenneth Boyer[13], A. Gwendolyn Noble[2], Charles N. Swisher[2], Peter T. Heydemann[13], Peter Rabiah[14], Shawn Withers[1], Patricia Soteropoulos[15], Leroy Hood[5], and Rima McLeod[1*]

[1]The University of Chicago, Chicago, IL 60637
[2]Northwestern University, Feinberg School of Medicine, Chicago, IL 60611
[3]BrainMicro LLC, New Haven, CT 06511
[4]J Craig Venter Institute, Rockville, MD 20850
[5]Institute of Systems Biology, Seattle, WA 98109
[6]University of Strathclyde, Glasgow G1 1XQ, United Kingdom
[7]Genome Quebec, Montréal, QC H3B 1S6, Canada; McGill University, Montréal, QC H3A 0G4, Canada
[8]Cambridge Institute for Medical Research and Department of Pathology, University of Cambridge, Cambridge CB2 1QP, United Kingdom
[9]Telethon Kids Institute, The University of Western Australia, Perth, Australia
[10]University of Illinois-Chicago, Chicago, IL 60607
[11]California Pacific Medical Center, San Francisco, CA 94114
[12]JM USDA Human Nutrition Research Center on Aging, Tufts University, Boston, MA 02111
[13]Rush University Medical Center, Chicago, IL 60612
[14]Northshore University Health System, Evanston, IL 60201
[15]Rutgers University, Newark, New Jersey 07101

[+] Equal contributions

* To whom Correspondence should be addressed:
Rima McLeod, M.D.
rmcleod@uchicago.edu

Co-Corresponding Authors:
Huân M. Ngô (h-ngo@northwestern.edu)
Hernan Lorenzi, PhD (hlorenzi@jcvi.org)
Kai Wang, PhD (kai.wang@systemsbiology.org)
T.K. Kim, MS (tkim@systemsbiology.org)

Current Addresses:
FLH, IBEHR School of Science and Sport, University of the West of Scotland, Paisley, PA1 2BE, UK

References 51-192

51 Shen, S. *et al.* rMATS: robust and flexible detection of differential alternative splicing from replicate RNA-Seq data. *Proc Natl Acad Sci U S A* **111**, E5593-5601, doi:10.1073/pnas.1419161111 (2014).

52 Lausted, C. *et al.* Systems approach to neurodegenerative disease biomarker discovery. *Annu Rev Pharmacol Toxicol* **54**, 457-481, doi:10.1146/annurev-pharmtox-011613-135928 (2014).

53 Franceschini, A. *et al.* STRING v9.1: protein-protein interaction networks, with increased coverage and integration. *Nucleic Acids Res* **41**, D808-815, doi:10.1093/nar/gks1094 (2013).

54 Gonzalez-Perez, O. *et al.* Immunological regulation of neurogenic niches in the adult brain. *Neuroscience* **226**, 270-281, doi:10.1016/j.neuroscience.2012.08.053 (2012).

55 Harris, T. H. *et al.* Generalized Levy walks and the role of chemokines in migration of effector CD8+ T cells. *Nature* **486**, 545-548, doi:10.1038/nature11098 (2012).

56 Vyas, A. Mechanisms of Host Behavioral Change in Toxoplasma gondii Rodent Association. *PLoS Pathog* **11**, e1004935, doi:10.1371/journal.ppat.1004935 (2015).

57 Tang, Y., Bao, J. S., Su, J. H. & Huang, W. MicroRNA-139 modulates Alzheimer's-associated pathogenesis in SAMP8 mice by targeting cannabinoid receptor type 2. *Genet Mol Res* **16**, doi:10.4238/gmr16019166 (2017).

58 Hebert, S. S. *et al.* Loss of microRNA cluster miR-29a/b-1 in sporadic Alzheimer's disease correlates with increased BACE1/beta-secretase expression. *Proc Natl Acad Sci U S A* **105**, 6415-6420, doi:10.1073/pnas.0710263105 (2008).

59 Shioya, M. *et al.* Aberrant microRNA expression in the brains of neurodegenerative diseases: miR-29a decreased in Alzheimer disease brains targets neurone navigator 3. *Neuropathol Appl Neurobiol* **36**, 320-330, doi:10.1111/j.1365-2990.2010.01076.x (2010).

60 Nelson, P. T. & Wang, W. X. MiR-107 is reduced in Alzheimer's disease brain neocortex: validation study. *J Alzheimers Dis* **21**, 75-79, doi:10.3233/JAD-2010-091603 (2010).

61 Wang, W. X., Huang, Q., Hu, Y., Stromberg, A. J. & Nelson, P. T. Patterns of microRNA expression in normal and early Alzheimer's disease human temporal cortex: white matter versus gray matter. *Acta Neuropathol* **121**, 193-205, doi:10.1007/s00401-010-0756-0 (2011).

62 Johnson, R. *et al.* A microRNA-based gene dysregulation pathway in Huntington's disease. *Neurobiol Dis* **29**, 438-445, doi:10.1016/j.nbd.2007.11.001 (2008).

63 Xia, H. *et al.* MiR-218 sensitizes glioma cells to apoptosis and inhibits tumorigenicity by regulating ECOP-mediated suppression of NF-kappaB activity. *Neuro Oncol* **15**, 413-422, doi:10.1093/neuonc/nos296 (2013).

64 Zhang, X. *et al.* Up-regulated microRNA-143 transcribed by nuclear factor kappa B enhances hepatocarcinoma metastasis by repressing fibronectin expression. *Hepatology* **50**, 490-499, doi:10.1002/hep.23008 (2009).

65 Borralho, P. M. *et al.* MicroRNA-143 reduces viability and increases sensitivity to 5-fluorouracil in HCT116 human colorectal cancer cells. *FEBS J* **276**, 6689-6700, doi:10.1111/j.1742-4658.2009.07383.x (2009).

66 Kluiver, J. *et al.* Regulation of pri-microRNA BIC transcription and processing in Burkitt lymphoma. *Oncogene* **26**, 3769-3776, doi:10.1038/sj.onc.1210147 (2007).

67 Chen, R. *et al.* Regulation of IKKbeta by miR-199a affects NF-kappaB activity in ovarian cancer cells. *Oncogene* **27**, 4712-4723, doi:10.1038/onc.2008.112 (2008).

68 Young, M. R., Santhanam, A. N., Yoshikawa, N. & Colburn, N. H. Have tumor suppressor PDCD4 and its counteragent oncogenic miR-21 gone rogue? *Mol Interv* **10**, 76-79, doi:10.1124/mi.10.2.5 (2010).

69 Sheedy, F. J. *et al.* Negative regulation of TLR4 via targeting of the proinflammatory tumor suppressor PDCD4 by the microRNA miR-21. *Nat Immunol* **11**, 141-147, doi:10.1038/ni.1828 (2010).

70 Li, T. *et al.* MicroRNAs modulate the noncanonical transcription factor NF-kappaB pathway by regulating expression of the kinase IKKalpha during macrophage differentiation. *Nat Immunol* **11**, 799-805, doi:10.1038/ni.1918 (2010).

71 Iliopoulos, D., Jaeger, S. A., Hirsch, H. A., Bulyk, M. L. & Struhl, K. STAT3 activation of miR-21 and miR-181b-1 via PTEN and CYLD are part of the epigenetic switch linking inflammation to cancer. *Mol Cell* **39**, 493-506, doi:10.1016/j.molcel.2010.07.023 (2010).

72 Pfeffer, M., Gispert, S., Auburger, G., Wicht, H. & Korf, H. W. Impact of Ataxin-2 knock out on circadian locomotor behavior and PER immunoreaction in the SCN of mice. *Chronobiol Int* **34**, 129-137, doi:10.1080/07420528.2016.1245666 (2017).

73 Kaehler, C. *et al.* Ataxin-2-like is a regulator of stress granules and processing bodies. *PLoS One* **7**, e50134, doi:10.1371/journal.pone.0050134 (2012).

74 Fernandez, E. & Mallette, F. A. The Rise of FXR1: Escaping Cellular Senescence in Head and Neck Squamous Cell Carcinoma. *PLoS Genet* **12**, e1006344, doi:10.1371/journal.pgen.1006344 (2016).

75 Papandreou, A. & Gissen, P. Diagnostic workup and management of patients with suspected Niemann-Pick type C disease. *Ther Adv Neurol Disord* **9**, 216-229, doi:10.1177/1756285616635964 (2016).

76 Hu, Y. H. *et al.* WDFY1 mediates TLR3/4 signaling by recruiting TRIF. *EMBO Rep* **16**, 447-455, doi:10.15252/embr.201439637 (2015).

77 Hu, M. C. *et al.* Protein phosphatase X interacts with c-Rel and stimulates c-Rel/nuclear factor kappaB activity. *J Biol Chem* **273**, 33561-33565 (1998).

78 Du, J. *et al.* Toxoplasma gondii virulence factor ROP18 inhibits the host NF-kappaB pathway by promoting p65 degradation. *J Biol Chem* **289**, 12578-12592, doi:10.1074/jbc.M113.544718 (2014).

79 Theodoropoulou, M., Reincke, M., Fassnacht, M. & Komada, M. Decoding the genetic basis of Cushing's disease: USP8 in the spotlight. *Eur J Endocrinol* **173**, M73-83, doi:10.1530/EJE-15-0320 (2015).
80 Durcan, T. M. *et al.* USP8 regulates mitophagy by removing K6-linked ubiquitin conjugates from parkin. *EMBO J* **33**, 2473-2491, doi:10.15252/embj.201489729 (2014).
81 McLelland, G. L., Soubannier, V., Chen, C. X., McBride, H. M. & Fon, E. A. Parkin and PINK1 function in a vesicular trafficking pathway regulating mitochondrial quality control. *EMBO J* **33**, 282-295, doi:10.1002/embj.201385902 (2014).
82 Sartori, S. *et al.* Angelman syndrome due to a novel splicing mutation of the UBE3A gene. *J Child Neurol* **23**, 912-915, doi:10.1177/0883073808316367 (2008).
83 Samaco, R. C., Hogart, A. & LaSalle, J. M. Epigenetic overlap in autism-spectrum neurodevelopmental disorders: MECP2 deficiency causes reduced expression of UBE3A and GABRB3. *Hum Mol Genet* **14**, 483-492, doi:10.1093/hmg/ddi045 (2005).
84 Chang, R. & Wang, E. Mouse translation elongation factor eEF1A-2 interacts with Prdx-I to protect cells against apoptotic death induced by oxidative stress. *J Cell Biochem* **100**, 267-278, doi:10.1002/jcb.20969 (2007).
85 Abbas, W., Kumar, A. & Herbein, G. The eEF1A Proteins: At the Crossroads of Oncogenesis, Apoptosis, and Viral Infections. *Front Oncol* **5**, 75, doi:10.3389/fonc.2015.00075 (2015).
86 Tsuyama, T., Tsubouchi, A., Usui, T., Imamura, H. & Uemura, T. Mitochondrial dysfunction induces dendritic loss via eIF2alpha phosphorylation. *J Cell Biol* **216**, 815-834, doi:10.1083/jcb.201604065 (2017).
87 Concepcion, C. P., Bonetti, C. & Ventura, A. The microRNA-17-92 family of microRNA clusters in development and disease. *Cancer J* **18**, 262-267, doi:10.1097/PPO.0b013e318258b60a (2012).
88 Srikanth, P. *et al.* Genomic DISC1 Disruption in hiPSCs Alters Wnt Signaling and Neural Cell Fate. *Cell Rep* **12**, 1414-1429, doi:10.1016/j.celrep.2015.07.061 (2015).
89 Zeiner, G. M., Norman, K. L., Thomson, J. M., Hammond, S. M. & Boothroyd, J. C. *Toxoplasma gondii* infection specifically increases the levels of key host microRNAs. *PLoS One* **5**, e8742, doi:10.1371/journal.pone.0008742 (2010).
90 Sonntag, K. C., Woo, T. U. & Krichevsky, A. M. Converging miRNA functions in diverse brain disorders: a case for miR-124 and miR-126. *Exp Neurol* **235**, 427-435, doi:10.1016/j.expneurol.2011.11.035 (2012).
91 Harold, D. *et al.* Genome-wide association study identifies variants at CLU and PICALM associated with Alzheimer's disease. *Nat Genet* **41**, 1088-1093, doi:10.1038/ng.440 (2009).
92 Lambert, J. C. *et al.* Genome-wide association study identifies variants at CLU and CR1 associated with Alzheimer's disease. *Nat Genet* **41**, 1094-1099, doi:10.1038/ng.439 (2009).
93 Schrijvers, E. M., Koudstaal, P. J., Hofman, A. & Breteler, M. M. Plasma clusterin and the risk of Alzheimer disease. *JAMA* **305**, 1322-1326, doi:10.1001/jama.2011.381 (2011).
94 Tennent, G. A., Lovat, L. B. & Pepys, M. B. Serum amyloid P component prevents proteolysis of the amyloid fibrils of Alzheimer disease and systemic amyloidosis. *Proc Natl Acad Sci U S A* **92**, 4299-4303 (1995).
95 Zhang, Y., McLaughlin, R., Goodyer, C. & LeBlanc, A. Selective cytotoxicity of intracellular amyloid beta peptide1-42 through p53 and Bax in cultured primary human neurons. *J Cell Biol* **156**, 519-529, doi:10.1083/jcb.200110119 (2002).
96 Baribeau, D. A. & Anagnostou, E. Oxytocin and vasopressin: linking pituitary neuropeptides and their receptors to social neurocircuits. *Front Neurosci* **9**, 335, doi:10.3389/fnins.2015.00335 (2015).
97 Jacob, S. *et al.* Association of the oxytocin receptor gene (OXTR) in Caucasian children and adolescents with autism. *Neurosci Lett* **417**, 6-9, doi:10.1016/j.neulet.2007.02.001 (2007).
98 Wermter, A. K. *et al.* Evidence for the involvement of genetic variation in the oxytocin receptor gene (OXTR) in the etiology of autistic disorders on high-functioning level. *Am J Med Genet B Neuropsychiatr Genet* **153B**, 629-639, doi:10.1002/ajmg.b.31032 (2010).
99 Tyzio, R. *et al.* Maternal oxytocin triggers a transient inhibitory switch in GABA signaling in the fetal brain during delivery. *Science* **314**, 1788-1792, doi:10.1126/science.1133212 (2006).
100 Kamphuis, W. *et al.* GFAP and vimentin deficiency alters gene expression in astrocytes and microglia in wild-type mice and changes the transcriptional response of reactive glia in mouse model for Alzheimer's disease. *Glia* **63**, 1036-1056, doi:10.1002/glia.22800 (2015).
101 Lambert, J. C. *et al.* Meta-analysis of 74,046 individuals identifies 11 new susceptibility loci for Alzheimer's disease. *Nat Genet* **45**, 1452-1458, doi:10.1038/ng.2802 (2013).
102 Weber, J. J., Sowa, A. S., Binder, T. & Hubener, J. From pathways to targets: understanding the mechanisms behind polyglutamine disease. *Biomed Res Int* **2014**, 701758, doi:10.1155/2014/701758 (2014).
103 Kumar, D. K. *et al.* Amyloid-beta peptide protects against microbial infection in mouse and worm models of Alzheimer's disease. *Sci Transl Med* **8**, 340ra372, doi:10.1126/scitranslmed.aaf1059 (2016).
104 Yamamoto, M. *et al.* ATF6beta is a host cellular target of the *Toxoplasma gondii* virulence factor ROP18. *J Exp Med* **208**, 1533-1546, doi:10.1084/jem.20101660 (2011).
105 Woo, C. W. *et al.* Adaptive suppression of the ATF4-CHOP branch of the unfolded protein response by toll-like receptor signalling. *Nat Cell Biol* **11**, 1473-1480, doi:10.1038/ncb1996 (2009).
106 Hetz, C. The unfolded protein response: controlling cell fate decisions under ER stress and beyond. *Nat Rev Mol Cell Biol* **13**, 89-102, doi:10.1038/nrm3270 (2012).
107 Blader, I. J., Manger, I. D. & Boothroyd, J. C. Microarray analysis reveals previously unknown changes in *Toxoplasma gondii*-infected human cells. *J Biol Chem* **276**, 24223-24231, doi:10.1074/jbc.M100951200 (2001).

108 Benevento, J. D. *et al.* Toxoplasmosis-associated neovascular lesions treated successfully with ranibizumab and antiparasitic therapy. *Arch Ophthalmol* **126**, 1152-1156, doi:10.1001/archopht.126.8.1152 (2008).
109 Muniz-Feliciano, L. *et al.* *Toxoplasma gondii*-induced activation of EGFR prevents autophagy protein-mediated killing of the parasite. *PLoS Pathog* **9**, e1003809, doi:10.1371/journal.ppat.1003809 (2013).
110 Spear, W. *et al.* The host cell transcription factor hypoxia-inducible factor 1 is required for *Toxoplasma gondii* growth and survival at physiological oxygen levels. *Cell Microbiol* **8**, 339-352, doi:10.1111/j.1462-5822.2005.00628.x (2006).
111 Franco, M., Shastri, A. J. & Boothroyd, J. C. Infection by *Toxoplasma gondii* specifically induces host c-Myc and the genes this pivotal transcription factor regulates. *Eukaryot Cell* **13**, 483-493, doi:10.1128/EC.00316-13 (2014).
112 Ong, Y. C., Reese, M. L. & Boothroyd, J. C. *Toxoplasma* rhoptry protein 16 (ROP16) subverts host function by direct tyrosine phosphorylation of STAT6. *J Biol Chem* **285**, 28731-28740, doi:10.1074/jbc.M110.112359 (2010).
113 Morgado, P., Ong, Y. C., Boothroyd, J. C. & Lodoen, M. B. *Toxoplasma gondii* induces B7-2 expression through activation of JNK signal transduction. *Infect Immun* **79**, 4401-4412, doi:10.1128/IAI.05562-11 (2011).
114 Wagner, J. P., Black, I. B. & DiCicco-Bloom, E. Stimulation of neonatal and adult brain neurogenesis by subcutaneous injection of basic fibroblast growth factor. *J Neurosci* **19**, 6006-6016 (1999).
115 Ohkubo, Y., Uchida, A. O., Shin, D., Partanen, J. & Vaccarino, F. M. Fibroblast growth factor receptor 1 is required for the proliferation of hippocampal progenitor cells and for hippocampal growth in mouse. *J Neurosci* **24**, 6057-6069, doi:10.1523/JNEUROSCI.1140-04.2004 (2004).
116 Paik, J. H. *et al.* FoxOs cooperatively regulate diverse pathways governing neural stem cell homeostasis. *Cell Stem Cell* **5**, 540-553, doi:10.1016/j.stem.2009.09.013 (2009).
117 Sanchez-Ramos, J. *et al.* Granulocyte colony stimulating factor decreases brain amyloid burden and reverses cognitive impairment in Alzheimer's mice. *Neuroscience* **163**, 55-72, doi:10.1016/j.neuroscience.2009.05.071 (2009).
118 Delli Carri, A. *et al.* Human pluripotent stem cell differentiation into authentic striatal projection neurons. *Stem Cell Rev* **9**, 461-474, doi:10.1007/s12015-013-9441-8 (2013).
119 Le Belle, J. E. *et al.* Proliferative neural stem cells have high endogenous ROS levels that regulate self-renewal and neurogenesis in a PI3K/Akt-dependant manner. *Cell Stem Cell* **8**, 59-71, doi:10.1016/j.stem.2010.11.028 (2011).
120 Golestaneh, N. & Mishra, B. TGF-beta, neuronal stem cells and glioblastoma. *Oncogene* **24**, 5722-5730, doi:10.1038/sj.onc.1208925 (2005).
121 Jaeger, I. *et al.* Temporally controlled modulation of FGF/ERK signaling directs midbrain dopaminergic neural progenitor fate in mouse and human pluripotent stem cells. *Development* **138**, 4363-4374, doi:10.1242/dev.066746 (2011).
122 Maire, C. L. *et al.* Pten loss in Olig2 expressing neural progenitor cells and oligodendrocytes leads to interneuron dysplasia and leukodystrophy. *Stem Cells* **32**, 313-326, doi:10.1002/stem.1590 (2014).
123 Shu, J. *et al.* Nedd8 targets ubiquitin ligase Smurf2 for neddylation and promote its degradation. *Biochem Biophys Res Commun* **474**, 51-56, doi:10.1016/j.bbrc.2016.04.058 (2016).
124 Cavadini, S. *et al.* Cullin-RING ubiquitin E3 ligase regulation by the COP9 signalosome. *Nature* **531**, 598-603, doi:10.1038/nature17416 (2016).
125 Swatek, K. N. & Komander, D. Ubiquitin modifications. *Cell Res* **26**, 399-422, doi:10.1038/cr.2016.39 (2016).
126 Poirotte, C. *et al.* Morbid attraction to leopard urine in *Toxoplasma*-infected chimpanzees. *Curr Biol* **26**, R98-99, doi:10.1016/j.cub.2015.12.020 (2016).
127 Fan, W. *et al.* Transcriptional profile of SH-SY5Y human neuroblastoma cells transfected by Toxoplasma rhoptry protein 16. *Mol Med Rep*, doi:10.3892/mmr.2016.5758 (2016).
128 Behnke, M. S., Dubey, J. P. & Sibley, L. D. Genetic Mapping of Pathogenesis Determinants in Toxoplasma gondii. *Annu Rev Microbiol* **70**, 63-81, doi:10.1146/annurev-micro-091014-104353 (2016).
129 Knoll, L. J. Functional Analysis of the Rhoptry Kinome during Chronic Toxoplasma gondii Infection. *MBio* **7**, doi:10.1128/mBio.00842-16 (2016).
130 Pernas, L., Ramirez, R., Holmes, T. H., Montoya, J. G. & Boothroyd, J. C. Immune profiling of pregnant Toxoplasma-infected US and Colombia patients reveals surprising impacts of infection on peripheral blood cytokines. *J Infect Dis* **210**, 923-931, doi:10.1093/infdis/jiu189 (2014).
131 Ngoungou, E. B., Bhalla, D., Nzoghe, A., Darde, M. L. & Preux, P. M. Toxoplasmosis and epilepsy--systematic review and meta analysis. *PLoS Negl Trop Dis* **9**, e0003525, doi:10.1371/journal.pntd.0003525 (2015).
132 Legido, A. & Katsetos, C. D. Experimental studies in epilepsy: immunologic and inflammatory mechanisms. *Semin Pediatr Neurol* **21**, 197-206, doi:10.1016/j.spen.2014.10.001 (2014).
133 Mawhinney, L. J., de Rivero Vaccari, J. P., Dale, G. A., Keane, R. W. & Bramlett, H. M. Heightened inflammasome activation is linked to age-related cognitive impairment in Fischer 344 rats. *BMC Neurosci* **12**, 123, doi:10.1186/1471-2202-12-123 (2011).
134 Pestov, D. G., Strezoska, Z. & Lau, L. F. Evidence of p53-dependent cross-talk between ribosome biogenesis and the cell cycle: effects of nucleolar protein Bop1 on G(1)/S transition. *Mol Cell Biol* **21**, 4246-4255, doi:10.1128/MCB.21.13.4246-4255.2001 (2001).
135 Gauthier, C. *et al.* Symptoms of depression and anxiety in anorexia nervosa: links with plasma tryptophan and serotonin metabolism. *Psychoneuroendocrinology* **39**, 170-178, doi:10.1016/j.psyneuen.2013.09.009 (2014).
136 Estes, M. L. & McAllister, A. K. Maternal TH17 cells take a toll on baby's brain. *Science* **351**, 919-920, doi:10.1126/science.aaf2850 (2016).

137 Li, Y. I. *et al.* RNA splicing is a primary link between genetic variation and disease. *Science* **352**, 600-604, doi:10.1126/science.aad9417 (2016).
138 Vittecoq, M. *et al.* Brain cancer mortality rates increase with *Toxoplasma gondii* seroprevalence in France. *Infect Genet Evol* **12**, 496-498, doi:10.1016/j.meegid.2012.01.013 (2012).
139 Torrey, E. F., Bartko, J. J. & Yolken, R. H. Toxoplasma gondii and other risk factors for schizophrenia: an update. *Schizophr Bull* **38**, 642-647, doi:10.1093/schbul/sbs043 (2012).
140 Prandovszky, E. *et al.* The neurotropic parasite Toxoplasma gondii increases dopamine metabolism. *PLoS One* **6**, e23866, doi:10.1371/journal.pone.0023866 (2011).
141 Martin, H. L. *et al.* Effect of parasitic infection on dopamine biosynthesis in dopaminergic cells. *Neuroscience* **306**, 50-62, doi:10.1016/j.neuroscience.2015.08.005 (2015).
142 Costa, C. *et al.* Epilepsy, amyloid-beta, and D1 dopamine receptors: a possible pathogenetic link? *Neurobiol Aging* **48**, 161-171, doi:10.1016/j.neurobiolaging.2016.08.025 (2016).
143 Sauer, T. C. *et al.* Primary intraocular (retinal) lymphoma after ocular toxoplasmosis. *Retin Cases Brief Rep* **4**, 160-163, doi:10.1097/ICB.0b013e3181ad3916 (2010).
144 Herbort, C. P. *et al.* Primary intraocular lymphoma: Possible role of Toxoplasma gondii in the lymphomagenesis. *Invest Ophth Vis Sci* **43**, U1212-U1212 (2002).
145 McLeod, R., Estes, R. G. & Cohen, H. Influence of Toxoplasma on manifestations of Moloney virus infections. *Trans R Soc Trop Med Hyg* **79**, 781-787 (1985).
146 Baird, J. R. *et al.* Immune-mediated regression of established B16F10 melanoma by intratumoral injection of attenuated Toxoplasma gondii protects against rechallenge. *Journal of immunology* **190**, 469-478, doi:10.4049/jimmunol.1201209 (2013).
147 Sanders, K. L., Fox, B. A. & Bzik, D. J. Attenuated Toxoplasma gondii therapy of disseminated pancreatic cancer generates long-lasting immunity to pancreatic cancer. *Oncoimmunology* **5**, e1104447, doi:10.1080/2162402X.2015.1104447 (2016).
148 Baird, J. R. *et al.* Avirulent Toxoplasma gondii generates therapeutic antitumor immunity by reversing immunosuppression in the ovarian cancer microenvironment. *Cancer Res* **73**, 3842-3851, doi:10.1158/0008-5472.CAN-12-1974 (2013).
149 Krahenbuhl, J. L. & Remington, J. S. Inhibition of target cell mitosis as a measure of the cytostatic effects of activated macrophages on tumor target cells. *Cancer Res* **37**, 3912-3916 (1977).
150 Ajani, J. A., Song, S., Hochster, H. S. & Steinberg, I. B. Cancer stem cells: the promise and the potential. *Semin Oncol* **42 Suppl 1**, S3-17, doi:10.1053/j.seminoncol.2015.01.001 (2015).
151 Kusbeci, O. Y., Miman, O., Yaman, M., Aktepe, O. C. & Yazar, S. Could Toxoplasma gondii have any role in Alzheimer disease? *Alzheimer Dis Assoc Disord* **25**, 1-3, doi:10.1097/WAD.0b013e3181f73bc2 (2011).
152 Nimgaonkar, V. L. *et al.* Temporal Cognitive Decline Associated With Exposure to Infectious Agents in a Population-based, Aging Cohort. *Alzheimer Dis Assoc Disord* **30**, 216-222, doi:10.1097/WAD.0000000000000133 (2016).
153 Mahami-Oskouei, M. *et al.* Toxoplasmosis and Alzheimer: can Toxoplasma gondii really be introduced as a risk factor in etiology of Alzheimer? *Parasitol Res* **115**, 3169-3174, doi:10.1007/s00436-016-5075-5 (2016).
154 Perry, C. E. *et al.* Seroprevalence and Serointensity of Latent Toxoplasma gondii in a Sample of Elderly Adults With and Without Alzheimer Disease. *Alzheimer Dis Assoc Disord* **30**, 123-126, doi:10.1097/WAD.0000000000000108 (2016).
155 Jung, B. K. *et al.* Toxoplasma gondii infection in the brain inhibits neuronal degeneration and learning and memory impairments in a murine model of Alzheimer's disease. *PLoS One* **7**, e33312, doi:10.1371/journal.pone.0033312 (2012).
156 Prandota, J. Possible link between Toxoplasma gondii and the anosmia associated with neurodegenerative diseases. *Am J Alzheimers Dis Other Demen* **29**, 205-214, doi:10.1177/1533317513517049 (2014).
157 Flegr, J., Lenochova, P., Hodny, Z. & Vondrova, M. Fatal attraction phenomenon in humans: cat odour attractiveness increased for toxoplasma-infected men while decreased for infected women. *PLoS Negl Trop Dis* **5**, e1389, doi:10.1371/journal.pntd.0001389 (2011).
158 Nance, J. P. *et al.* Chitinase dependent control of protozoan cyst burden in the brain. *PLoS Pathog* **8**, e1002990, doi:10.1371/journal.ppat.1002990 (2012).
159 McPhillie, M. *et al.* New paradigms for understanding and step changes in treating active and chronic, persistent apicomplexan infections. *Sci Rep* **6**, 29179, doi:10.1038/srep29179 (2016).
160 Reinartz, R. *et al.* Functional Subclone Profiling for Prediction of Treatment-Induced Intratumor Population Shifts and Discovery of Rational Drug Combinations in Human Glioblastoma. *Clin Cancer Res* **23**, 562-574, doi:10.1158/1078-0432.CCR-15-2089 (2017).
161 Ignatova, T. N. *et al.* Human cortical glial tumors contain neural stem-like cells expressing astroglial and neuronal markers in vitro. *Glia* **39**, 193-206, doi:10.1002/glia.10094 (2002).
162 Siebzehnrubl, F. A., Reynolds, B. A., Vescovi, A., Steindler, D. A. & Deleyrolle, L. P. The origins of glioma: E Pluribus Unum? *Glia* **59**, 1135-1147, doi:10.1002/glia.21143 (2011).
163 Siebzehnrubl, F. A. *et al.* The ZEB1 pathway links glioblastoma initiation, invasion and chemoresistance. *EMBO Mol Med* **5**, 1196-1212, doi:10.1002/emmm.201302827 (2013).
164 Wang, S. *et al.* Neurogenic potential of progenitor cells isolated from postmortem human Parkinsonian brains. *Brain Res* **1464**, 61-72, doi:10.1016/j.brainres.2012.04.039 (2012).
165 Heneka, M. T. *et al.* Neuroinflammation in Alzheimer's disease. *Lancet Neurol* **14**, 388-405, doi:10.1016/S1474-4422(15)70016-5 (2015).

166 Silver, D. J. & Steindler, D. A. Common astrocytic programs during brain development, injury and cancer. *Trends Neurosci* **32**, 303-311, doi:10.1016/j.tins.2009.01.008 (2009).
167 Gabriel, E. *et al.* Recent Zika Virus Isolates Induce Premature Differentiation of Neural Progenitors in Human Brain Organoids. *Cell Stem Cell*, doi:10.1016/j.stem.2016.12.005 (2017).
168 Kukekov, V. G. *et al.* Multipotent stem/progenitor cells with similar properties arise from two neurogenic regions of adult human brain. *Exp Neurol* **156**, 333-344, doi:10.1006/exnr.1999.7028 (1999).
169 Eriksson, P. S. *et al.* Neurogenesis in the adult human hippocampus. *Nat Med* **4**, 1313-1317, doi:10.1038/3305 (1998).
170 Candelario, K. M. & Steindler, D. A. The role of extracellular vesicles in the progression of neurodegenerative disease and cancer. *Trends Mol Med* **20**, 368-374, doi:10.1016/j.molmed.2014.04.003 (2014).
171 Rajendran, L. *et al.* Alzheimer's disease beta-amyloid peptides are released in association with exosomes. *Proc Natl Acad Sci U S A* **103**, 11172-11177, doi:10.1073/pnas.0603838103 (2006).
172 Lietz, M., Hohl, M. & Thiel, G. RE-1 silencing transcription factor (REST) regulates human synaptophysin gene transcription through an intronic sequence-specific DNA-binding site. *Eur J Biochem* **270**, 2-9 (2003).
173 Coccaro, E. F. *et al. Toxoplasma gondii* infection: relationship with aggression in psychiatric subjects. *The Journal of clinical psychiatry* **77**, 334-341, doi:10.4088/JCP.14m09621 (2016).
174 Millar, J. K. *et al.* DISC1 and PDE4B are interacting genetic factors in schizophrenia that regulate cAMP signaling. *Science* **310**, 1187-1191, doi:10.1126/science.1112915 (2005).
175 Magill, S. T. *et al.* microRNA-132 regulates dendritic growth and arborization of newborn neurons in the adult hippocampus. *Proc Natl Acad Sci U S A* **107**, 20382-20387, doi:10.1073/pnas.1015691107 (2010).
176 Thirugnanam, S., Rout, N. & Gnanasekar, M. Possible role of *Toxoplasma gondii* in brain cancer through modulation of host microRNAs. *Infectious agents and cancer* **8**, 8, doi:10.1186/1750-9378-8-8 (2013).
177 Thomas, F. *et al.* Incidence of adult brain cancers is higher in countries where the protozoan parasite *Toxoplasma gondii* is common. *Biology letters* **8**, 101-103, doi:10.1098/rsbl.2011.0588 (2012).
178 Wang, Y. & Kasper, L. H. The role of microbiome in central nervous system disorders. *Brain Behav Immun* **38**, 1-12, doi:10.1016/j.bbi.2013.12.015 (2014).
179 Hassan, M. I., Naiyer, A. & Ahmad, F. Fragile histidine triad protein: structure, function, and its association with tumorogenesis. *J Cancer Res Clin Oncol* **136**, 333-350, doi:10.1007/s00432-009-0751-9 (2010).
180 Sanchis-Belenguer, R., Cuadrado-Mendez, L. & Ortiz Munoz, A. B. [Possible interactions between *Toxoplasma gondii* infection and the presence of carcinomas of female genitalia and the breast]. *Revista espanola de oncologia* **31**, 247-255 (1984).
181 Tan, J. Y. *et al.* Cross-talking noncoding RNAs contribute to cell-specific neurodegeneration in SCA7. *Nat Struct Mol Biol* **21**, 955-961, doi:10.1038/nsmb.2902 (2014).
182 Joscelyn, J. & Kasper, L. H. Digesting the emerging role for the gut microbiome in central nervous system demyelination. *Mult Scler* **20**, 1553-1559, doi:10.1177/1352458514541579 (2014).
183 Choi, G. B. *et al.* The maternal interleukin-17a pathway in mice promotes autism-like phenotypes in offspring. *Science* **351**, 933-939, doi:10.1126/science.aad0314 (2016).
184 Castanon, N., Luheshi, G. & Laye, S. Role of neuroinflammation in the emotional and cognitive alterations displayed by animal models of obesity. *Front Neurosci* **9**, 229, doi:10.3389/fnins.2015.00229 (2015).
185 Gamazon, E. R. *et al.* A gene-based association method for mapping traits using reference transcriptome data. *Nat Genet* **47**, 1091-1098, doi:10.1038/ng.3367 (2015).
186 Keidel, E. M., Dosch, D., Brunner, A., Kellermann, J. & Lottspeich, F. Evaluation of protein loading techniques and improved separation in OFFGEL isoelectric focusing. *Electrophoresis* **32**, 1659-1666, doi:10.1002/elps.201000544 (2011).
187 Warren, C. M., Geenen, D. L., Helseth, D. L., Jr., Xu, H. & Solaro, R. J. Sub-proteomic fractionation, iTRAQ, and OFFGEL-LC-MS/MS approaches to cardiac proteomics. *J Proteomics* **73**, 1551-1561, doi:10.1016/j.jprot.2010.03.016 (2010).
188 Xu, H. & Freitas, M. A. A mass accuracy sensitive probability based scoring algorithm for database searching of tandem mass spectrometry data. *BMC Bioinformatics* **8**, 133, doi:10.1186/1471-2105-8-133 (2007).
189 Grosse-Coosmann, F., Boehm, A. M. & Sickmann, A. Efficient analysis and extraction of MS/MS result data from Mascot result files. *BMC Bioinformatics* **6**, 290, doi:10.1186/1471-2105-6-290 (2005).
190 Thomas, S. & Bonchev, D. A survey of current software for network analysis in molecular biology. *Human genomics* **4**, 353-360 (2010).
191 Mirza, Z., Kamal, M. A., Buzenadah, A. M., Al-Qahtani, M. H. & Karim, S. Establishing genomic/transcriptomic links between Alzheimer's disease and type 2 diabetes mellitus by meta-analysis approach. *CNS & neurological disorders drug targets* **13**, 501-516 (2014).
192 Szklarczyk, D. *et al.* STRING v10: protein-protein interaction networks, integrated over the tree of life. *Nucleic Acids Res* **43**, D447-452, doi:10.1093/nar/gku1003 (2015).
